# Identification of C/EBPα as a novel target of the HPV8 E6 protein regulating miR-203 in human keratinocytes

**DOI:** 10.1371/journal.ppat.1006406

**Published:** 2017-06-22

**Authors:** Anna M. Marthaler, Marta Podgorska, Pascal Feld, Alina Fingerle, Katrin Knerr-Rupp, Friedrich Grässer, Hans Smola, Klaus Roemer, Elke Ebert, Yoo-Jin Kim, Rainer M. Bohle, Cornelia S. L. Müller, Jörg Reichrath, Thomas Vogt, Magdalena Malejczyk, Sławomir Majewski, Sigrun Smola

**Affiliations:** 1 Institute of Virology, Saarland University, Homburg/Saar, Germany; 2 Hartmann AG, Heidenheim, Germany; 3 Department of Dermatology, University of Cologne, Cologne, Germany; 4 Jose Carreras Center for Immune and Gene Therapy, Saarland University Medical Center, Homburg/Saar, Germany; 5 Institute of Pathology, Saarland University Medical Center, Homburg/Saar, Germany; 6 Department of Dermatology, Saarland University Medical Center, Homburg/Saar, Germany; 7 Diagnostic Laboratory of STDs, Department of Dermatology and Venereology, Medical University of Warsaw, Warsaw, Poland; 8 Department of Dermatology and Venereology, Medical University of Warsaw, Warsaw, Poland; University of Wisconsin-Madison, UNITED STATES

## Abstract

Patients suffering from Epidermodysplasia verruciformis (EV), a rare inherited skin disease, display a particular susceptibility to persistent infection with cutaneous genus beta-human papillomavirus (beta-HPV), such as HPV type 8. They have a high risk to develop non-melanoma skin cancer at sun-exposed sites. In various models evidence is emerging that cutaneous HPV E6 proteins disturb epidermal homeostasis and support carcinogenesis, however, the underlying mechanisms are not fully understood as yet. In this study we demonstrate that microRNA-203 (miR-203), a key regulator of epidermal proliferation and differentiation, is strongly down-regulated in HPV8-positive EV-lesions. We provide evidence that CCAAT/enhancer-binding protein α (C/EBPα), a differentiation-regulating transcription factor and suppressor of UV-induced skin carcinogenesis, directly binds the miR-203 gene within its hairpin region and thereby induces miR-203 transcription. Our data further demonstrate that the HPV8 E6 protein significantly suppresses this novel C/EBPα/mir-203-pathway. As a consequence, the miR-203 target ΔNp63α, a proliferation-inducing transcription factor, is up-regulated, while the differentiation factor involucrin is suppressed. HPV8 E6 specifically down-regulates C/EBPα but not C/EBPβ expression at the transcriptional level. As shown in knock-down experiments, C/EBPα is regulated by the acetyltransferase p300, a well-described target of cutaneous E6 proteins. Notably, p300 bound significantly less to the C/EBPα regulatory region in HPV8 E6 expressing keratinocytes than in control cells as demonstrated by chromatin immunoprecipitation. In situ analysis confirmed congruent suprabasal expression patterns of C/EBPα and miR-203 in non-lesional skin of EV-patients. In HPV8-positive EV-lesions both factors are potently down-regulated in vivo further supporting our in vitro data. In conclusion our study has unraveled a novel p300/C/EBPα/mir-203-dependent mechanism, by which the cutaneous HPV8 E6 protein may expand p63-positive cells in the epidermis of EV-patients and disturbs fundamental keratinocyte functions. This may drive HPV-mediated pathogenesis and may potentially also pave the way for skin carcinogenesis in EV-patients.

## Author summary

Cutaneous genus beta-HPV types infect skin keratinocytes. Their potential role in skin carcinogenesis, particularly in immunosuppressed patients, has become a major field of interest. Patients suffering from the rare genetic disorder Epidermodysplasia verruciformis (EV) are highly susceptible to persistent genus beta-HPV infection and have an increased risk to develop non-melanoma skin cancer at sun-exposed sites. Thus, EV serves as a valuable model disease for studying genus beta-HPV biology. Here, we demonstrate that in human HPV8-infected EV skin lesions, the ‘stemness-repressing’ microRNA-203 is strongly down-regulated. In contrast, cells expressing the miR-203-regulated ‘stemness-maintaining’ factor p63, are highly amplified. Notably, we identified the transcription factor C/EBPα, a well-known suppressor of UV-induced skin carcinogenesis, as a p300-dependent target of the HPV8-encoded E6 oncoprotein and as a critical inducer of miR-203 gene expression. Our data provide evidence for a novel p300/C/EBPα/miR-203-dependent pathway, which links HPV8 infection to the expansion of p63-positive cells in the epidermis of EV-patients. This may contribute to the beta-HPV-induced disturbance of epidermal homeostasis and pave the way for skin carcinogenesis.

## Introduction

Human papillomaviruses (HPV) are double-stranded non-enveloped DNA viruses that infect epithelial cells of skin or mucosa in a species-specific manner. They cause hyperproliferative lesions ranging from benign warts to invasive carcinoma. More than 180 HPV types are classified into five genera based on sequence homologies in the structural protein L1 [1]. The genus alpha mucosal high-risk (HR) HPV types, i.e. HPV16 and 18, have a well-established causal role in cervical carcinogenesis and they are involved in a significant proportion of other anogenital as well as oropharyngeal cancers [2]. In contrast, genus beta-HPV types infect cutaneous epithelium and their potential role in skin carcinogenesis particularly in immunosuppressed patients has become a major field of interest. A link between HPV infection and skin cancer was first demonstrated in patients suffering from Epidermodysplasia verruciformis (EV), a rare inherited skin disease. EV-patients display a particular susceptibility to productive and persistent infection with cutaneous genus beta-HPV and they have a high risk to develop non-melanoma skin cancer at sun-exposed sites [3, 4]. In EV-lesions, genus beta-HPVs strongly replicate and the viral genome is highly expressed [5, 6]. EV-cancers preferentially harbor HPV5 or HPV8 [7]. The functions of genus beta-HPV oncoproteins are, however, less well investigated than those of mucosal HPVs. Viral persistence in lesional skin of EV-patients is linked to the ‘virtual absence’ of Langerhans cells, specialized antigen-presenting cells normally residing in the epidermis [5]. The HPV8 E7 protein has been identified as a factor critical for viral immune evasion targeting a key pathway responsible for Langerhans cell attraction in the skin [5].

Studies in transgenic mice unraveled the E6 protein as the major driver of HPV8-induced skin carcinogenesis [8, 9]. Beta-HPV E6 proteins can suppress UV-induced apoptosis as well as DNA damage repair [10–12], stabilize p53 in response to genome-destabilizing events [13], interfere with Notch-signalling [14–17] and inhibit keratinocyte differentiation [15, 18]. Many of these functions depend on their ability to bind the acetyltransferase p300 [12, 13, 18]. The in vivo importance of the cutaneous PV E6-p300 interaction for tumorigenesis was highlighted in the cottontail rabbit PV (CRPV) model [19]. However, the mechanisms how the E6 protein contributes to beta-HPV-mediated disturbance of epidermal homeostasis are not yet completely understood.

HPV infects basal keratinocytes of stratifying epithelia. When basal cells become suprabasal, they lose their proliferative potential and undergo terminal differentiation. This process is governed by microRNA-203 (miR-203), which promotes epidermal differentiation and induces cell-cycle exit [20–22]. miR-203 is up-regulated in response to differentiation-inducing agents including Ca^2+^, Vitamin D3 or phorbol ester-induced protein kinase C (PKC) activity [21, 23]. A major target of miR-203 in suprabasal keratinocytes is the transcription factor p63, a member of the p53 family [20, 21]. The main isoform expressed in basal keratinocytes is the N-truncated isoform ΔNp63α lacking the transactivation domain [24]. This isoform is targeted by miR-203 in mammary epithelial cells mediating subversion of stem cell properties [25]. ΔNp63α plays a pivotal role in maintaining keratinocyte ‘stemness’ and proliferative capacity [26] and can suppress keratinocyte differentiation, i.e. expression of involucrin [27]. Its overexpression has been observed in squamous cell carcinomas of head and neck (HNSCC), lung and skin [28]. In case of HNSCCs this is associated with a poor prognosis and aggressive course of disease [29].

Notably, high levels of miR-203 are inhibitory to HPV amplification in differentiating keratinocytes indicating that inhibition of the miR-203 pathway is crucial for the viral life cycle [30]. In fact, for the mucosal HR-HPV types, it was reported that the E6, E7 and E5 proteins have the capacity to suppress miR-203 expression [30–32]. The HPV31 E7 protein was shown to down-regulate miR-203 expression upon phorbol ester induced differentiation [30]. The HPV16 E6 protein involves a p53-dependent mechanism [31].

So far, it has been unclear whether or not beta-HPV, which encode an E6 and E7 but no E5 gene (summarized in [33]), can impair miR-203 expression. In this study we show that the HPV8 E6 protein is a potent suppressor of miR-203, a key regulator of ΔNp63α expression, proliferation and differentiation in keratinocytes. We have identified the transcription factor CCAAT/enhancer-binding protein α (C/EBPα), a well-known driver of keratinocyte differentiation [34, 35], as a novel target of the HPV8 E6 protein regulating miR-203 expression. In situ stainings in EV-lesions strongly support our findings.

## Results

### miR-203 suppression and p63 up-regulation in lesional skin of EV-patients

**Fig 1:**
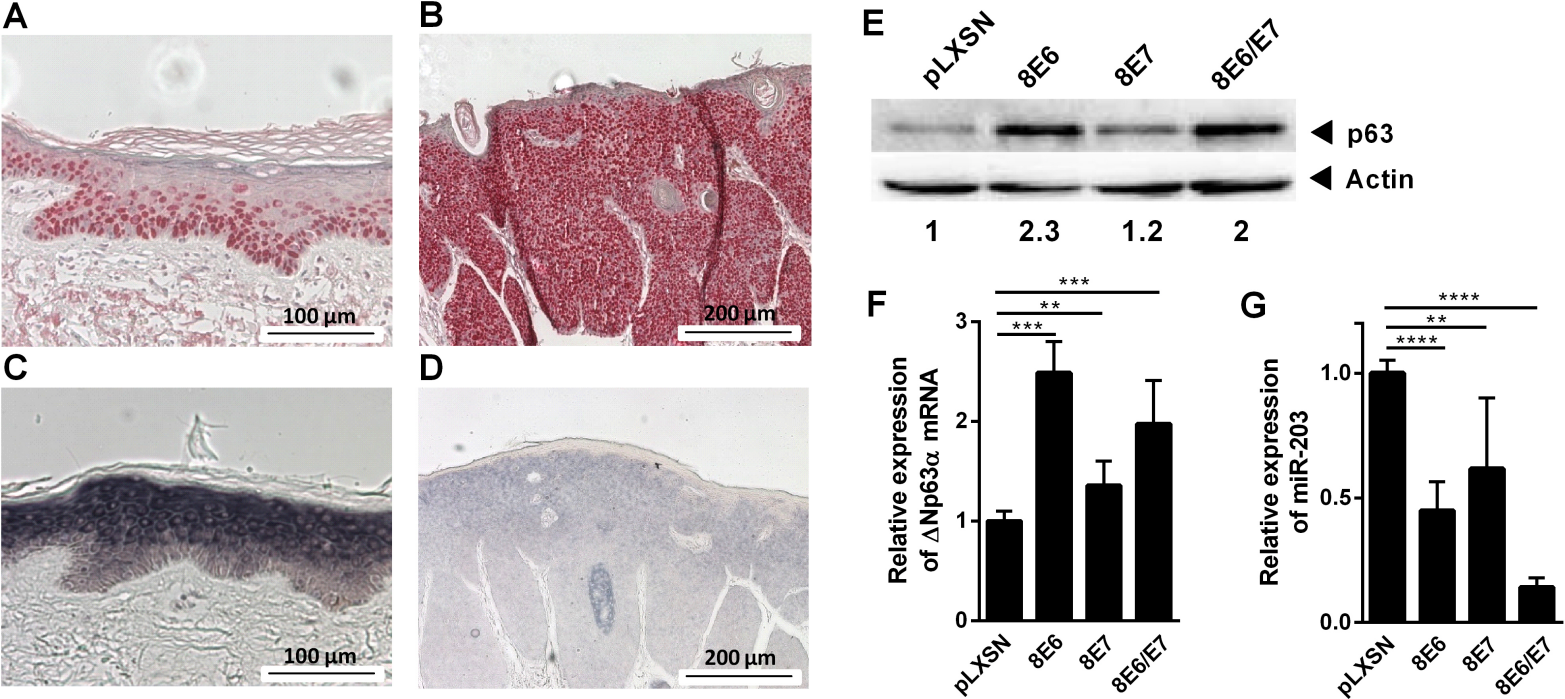
p63 and miR-203 regulation in EV-lesions and by HPV8 oncoproteins in NHK in vitro. Sections of non-lesional skin **(A, C)** and HPV8-positive lesional skin **(B, D)** from EV-patients were stained using antibodies against p63 (A, B) or probes against miR-203 (C, D). NHK stably expressing HPV8 E6, E7, E6/E7 or the corresponding pLXSN control cells were analyzed for **(E)** p63 protein expression by Western blot in relation to actin expression (shown is one experiment out of three), **(F)** ΔNp63α mRNA or **(G)** miR-203 expression by qRT-PCR. ΔNp63α mRNA was determined in relation to RPL13A, miR-203 level to RNU6B by the 2^ΔΔCt^ method. In (F, G) mean values ± SD from *n* ≥ 3 experiments performed in duplicates are shown. (**p<0.01, ***p<0.001, ****p<0.0001)

Since miR-203 and p63 are major regulators of epidermal homeostasis, which may be disturbed during genus beta-HPV infection in vivo, we compared expression patterns of both factors in non-lesional and HPV8-positive lesional skin of EV-patients. In non-lesional areas p63 was expressed in the nuclei of basal and parabasal keratinocytes (Fig 1A). As expected from findings in normal human skin [36], miR-203 displayed a complementary expression pattern and was detected in suprabasal spinous as well as granular layers but not in basal or parabasal keratinocytes (Fig 1C). Notably, in lesional skin of EV-patients p63-expressing cell layers were dramatically expanded and this corresponded to an almost complete absence of miR-203 (Fig 1B and 1D).

### HPV8 E6 down-regulates miR-203

To investigate whether HPV8 oncogenes contribute to the deregulation of both cellular factors, primary human keratinocytes (NHK) were engineered to express HPV8 E6 and/or E7 by retroviral gene transfer (S1A and S1B Fig). In HPV8 E6 expressing NHK, p63 was significantly up-regulated at protein and mRNA levels as shown by Western blot and ΔNp63α-specific qRT-PCR (Fig 1E and 1F). Correspondingly, in HPV8 E6 expressing keratinocytes miR-203 was significantly down-regulated (Fig 1G). HPV8 E7 alone had a less strong effect but enhanced the impact of E6, particularly on miR-203 suppression.

**Fig 2:**
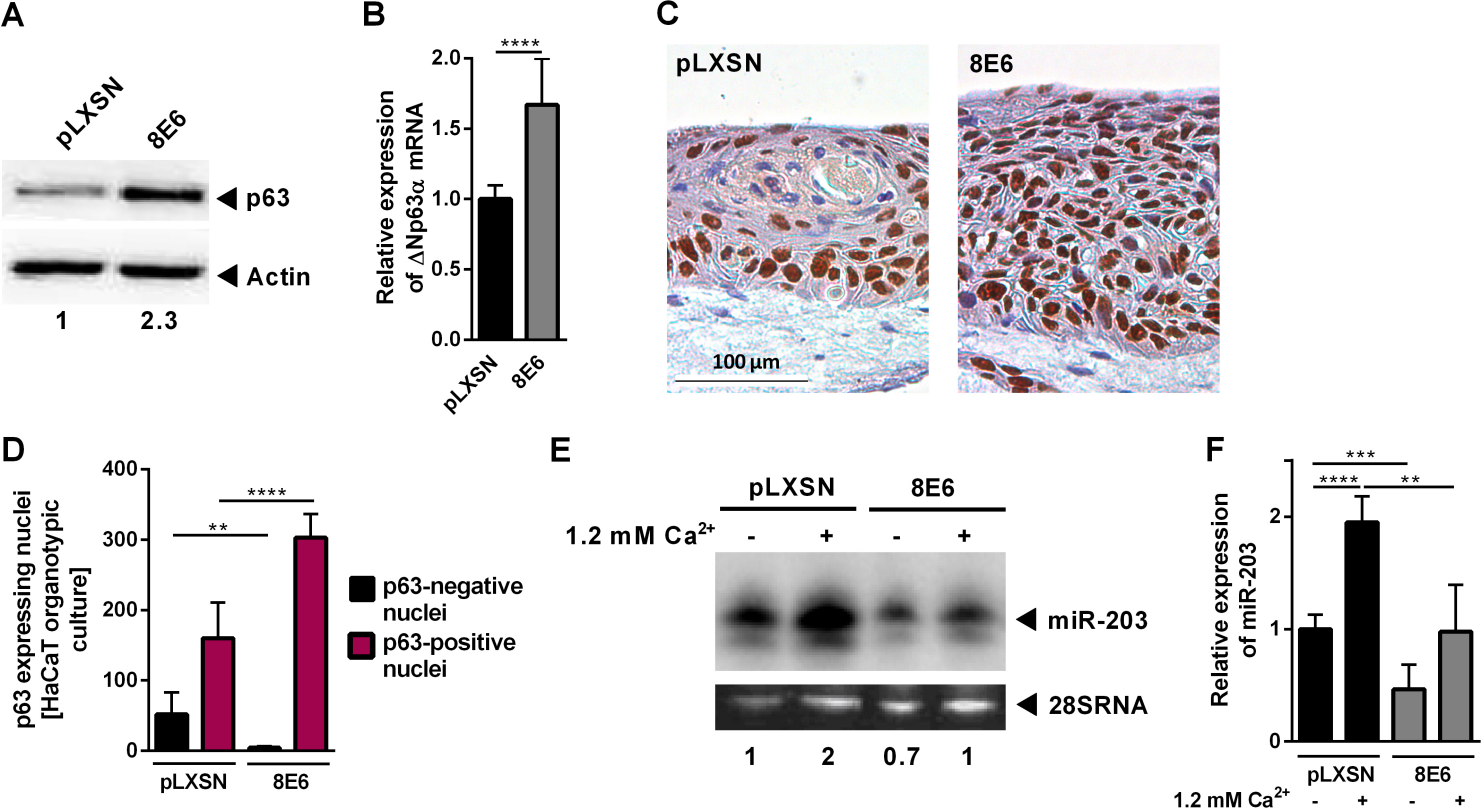
p63 and miR-203 expression are altered in HPV8 E6 expressing HaCaT cells. HaCaT cells stably expressing HPV8 E6 or corresponding pLXSN control cells were investigated in different experiments. **(A)** p63 protein expression was analyzed by Western blot in relation to actin (shown is one experiment out of three), **(B)** ΔNp63α mRNA expression by qRT-PCR in relation to RPL13A. **(C)** Organotypic cultures were stained for p63 expression (brown). **(D)** Three independent cultures were quantified for p63-positive and -negative nuclei (per microscopic field in randomly selected areas, 200x). **(E)** MiR-203 expression was determined after treatment with Ca^2+^ for 72 h by Northern blot. 28S RNA served as loading control. One experiment out of three is shown. **(F)** The same RNA as in (E) was used to analyze miR-203 levels by qRT-PCR. Expression levels were normalized on RNU6B by 2^ΔΔCt^. Mean values ± SD from *n* ≥ 3 experiments performed in duplicates are shown. (**p<0.01, ***p<0.001, ****p<0.0001)

HPV16 E6 can regulate miR-203 via p53 [31]. We therefore included the skin keratinocyte cell line HaCaT harboring mutated *TP53* genes in our study [37]. Interestingly, HaCaT cells expressing HPV8 E6 (S2A Fig), displayed significantly higher p63 protein and ΔNp63α mRNA levels than control cells similar to NHK (Fig 2A and 2B, S2B Fig).

Compared to control cultures, organotypic cultures with HPV8 E6 expressing HaCaT cells formed significantly more layers (as measured by the number of nuclei per microscopic field) and significantly more cells were found to express nuclear p63 similar to EV-lesions (Fig 2C and 2D).

To assess the impact of HPV8 E6 on miR-203 expression under differentiation conditions, we also stimulated HaCaT cells with Ca^2+^, a potent inducer of keratinocyte differentiation [38]. MiR-203 was up-regulated by 1.2 mM Ca^2+^ after 72 h as shown by Northern blot and qRT-PCR in control cells. In contrast, HPV8 E6 expressing HaCaT cells displayed lower constitutive miR-203 expression levels and Ca^2+^-mediated miR-203 induction was significantly suppressed (Fig 2E and 2F, S2C Fig).

These data indicated that HPV8 E6 exerts its inhibitory effects on miR-203 also in keratinocytes with mutated p53.

### HPV8 E6 engages the miR-203/p63-pathway to regulate key keratinocyte functions

**Fig 3:**
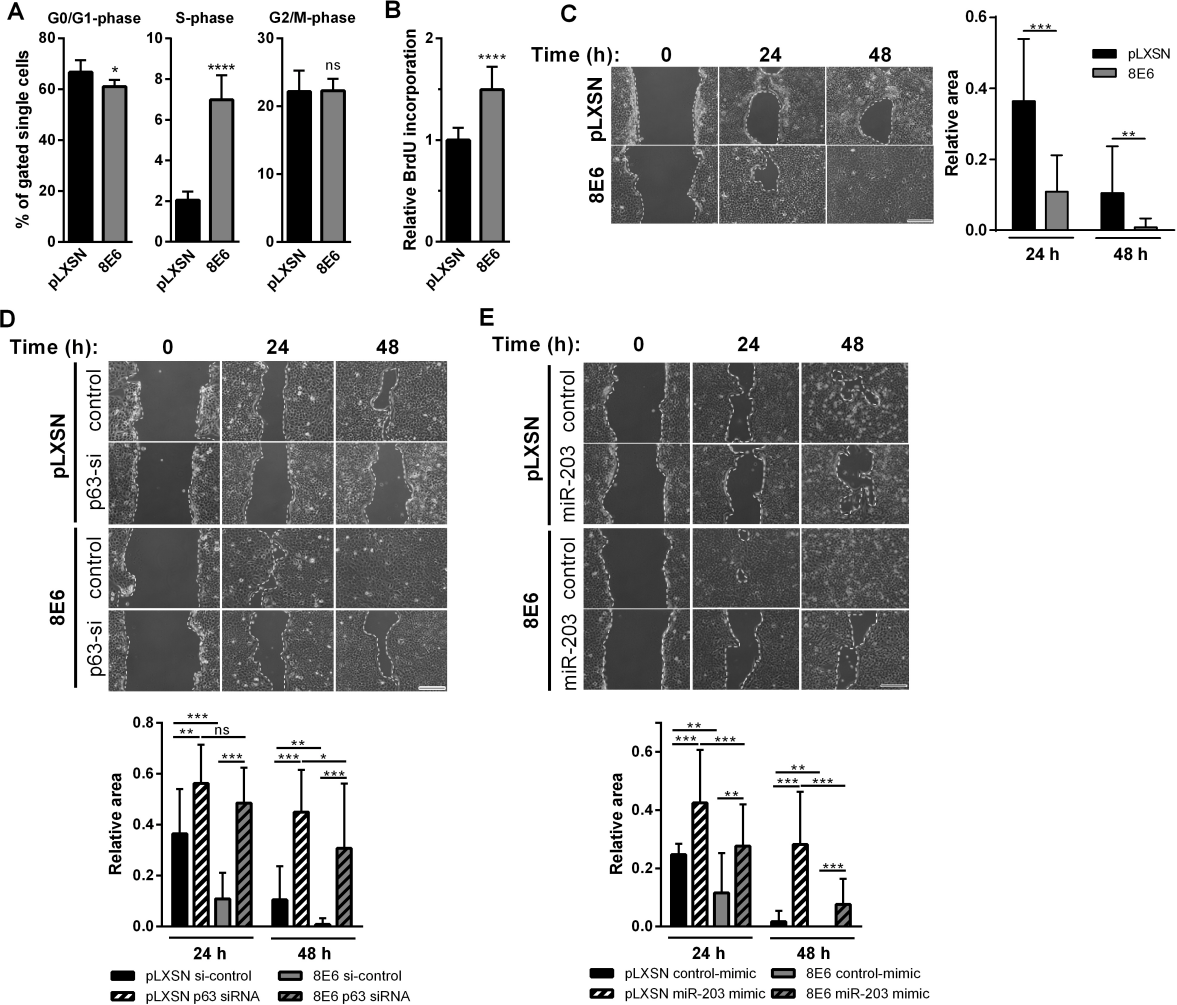
HPV8 E6 involves the miR203/p63-pathway to reprogram keratinocyte functions. **(A)** Cell cycle analysis of HPV8 E6 expressing and pLXSN control HaCaT cells by flow cytometry using propidium iodide staining. Shown are cell counts in the respective cell cycle phases in relation to the gated single cell population. **(B)** BrdU incorporation was measured in triplicates in HPV8 E6 expressing HaCaT cells and normalized to pLXSN-control. **(C)** HPV8 E6 expressing and pLXSN control HaCaT monolayer cultures were scratched. Pictures were taken at time points 0, 24 and 48 h. The area of the gap, indicated by dotted lines (left panel), was determined in relation to time point 0 h (right panel). HPV8 E6 expressing cells were scratched 24 h after 10 nM p63-siRNA (or si-control) **(D),** or 20 nM miR-203-mimic transfection (or control-mimic) **(E)** and analyzed as in (B). Scale bar: 200 μm. Mean values ± SD from *n* = 3 experiments are shown. (ns: not significant, *p<0.05, **p<0.01, ***p<0.001, ****p<0.0001)

We were then interested whether HPV8 E6 affects the cell cycle and proliferation. Cell cycle analysis by flow cytometry revealed that HPV8 E6 expression in asynchronously growing HaCaT cells resulted in a significant increase of cells in S-phase and a reduction of cells in G1 (Fig 3A), features critically regulated by p63 in keratinocytes [39]. Moreover, HPV8 E6 expressing HaCaT cells showed significantly higher BrdU incorporation than control cells indicating a significantly stronger rate of proliferation (Fig 3B). A similar finding was obtained in scratch assays measuring both proliferation and migration. Here, the introduced gap was closed significantly faster in HPV8 E6-expressing than in control cells (Fig 3C).

**Fig 4:**
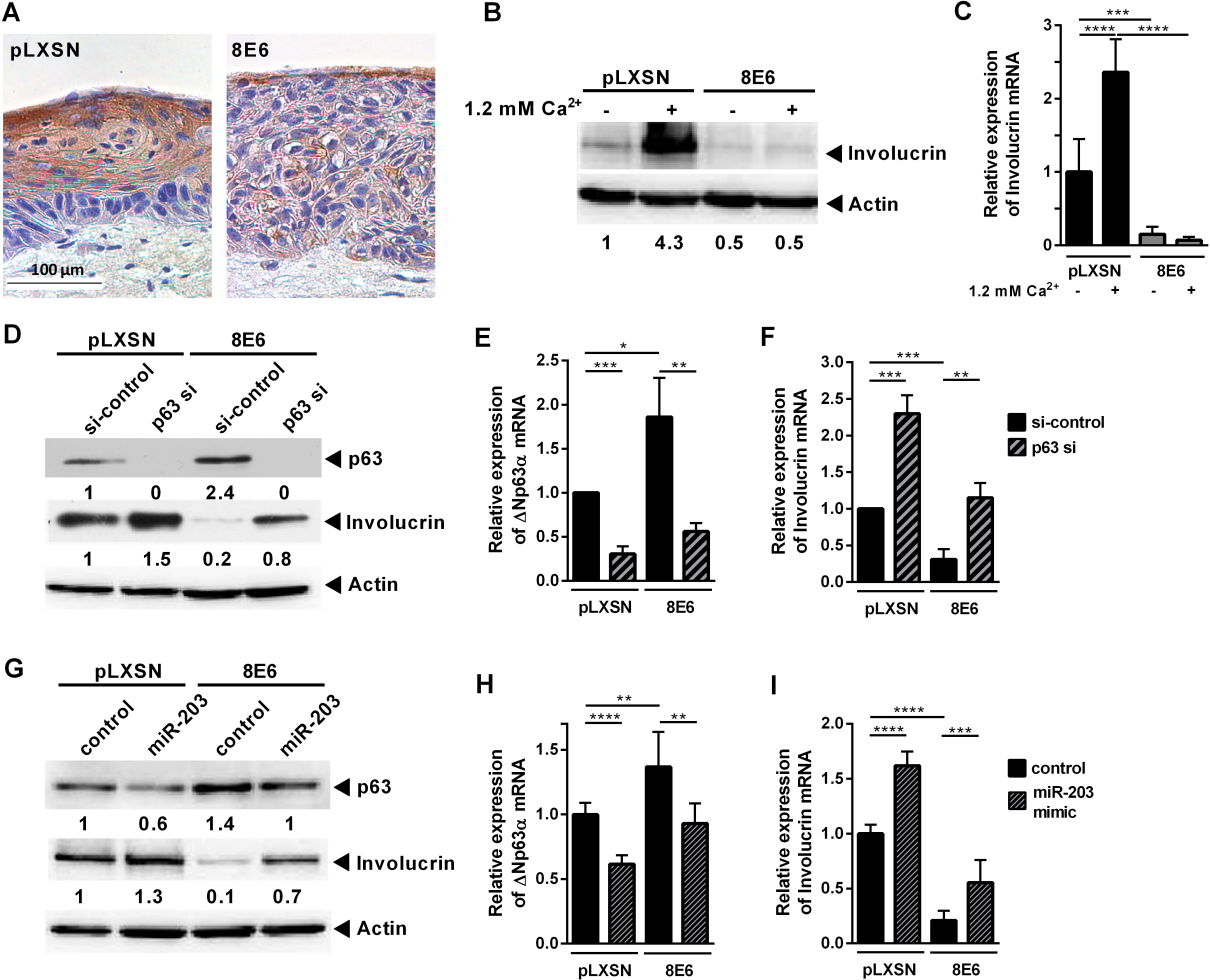
Differentiation suppression by HPV8 E6 involves the miR203/p63-pathway. Organotypic cultures generated from **(A)** HPV8 E6 expressing or pLXSN control HaCaT cells were stained for involucrin expression (brown). **(B,C)** HaCaT cells were stimulated with Ca^2+^ for 72 h. **(B)** Involucrin protein expression was determined by Western blot in relation to actin (shown is one experiment out of three), **(C)** involucrin mRNA expression by qRT-PCR in relation to RPL13A. **(D, E, F)** HPV8 E6 expressing and pLXSN control HaCaT cells were transfected with 10 nM p63 specific siRNA (or si-control). **(D)** 48 h post transfection, p63 and involucrin protein expression levels were determined by Western blot in relation to actin expression (shown is one experiment out of three), **(E)** ΔNp63α and **(F)** involucrin mRNA expression by qRT-PCR in relation to RPL13A. **(G, H, I)** HPV8 E6 expressing HaCaT cells were transfected with 20 nM hsa-miR-203-mimic (or control-mimic). 72 h post transfection cells were analyzed as in D-F. Mean values ± SD from *n* ≥ 3 experiments performed in duplicates are shown. (*p<0.05, **p<0.01, ***p<0.001, ****p<0.0001)

To explore a mechanistic role of p63 or miR-203, we knocked-down p63 or transfected an hsa-miR-203 mimic. p63 knock-down efficiency was 70% as quantified by RT-PCR (S3 Table). p63-specific siRNA strongly reduced p63 protein and ΔNp63α mRNA expression in control cells as well as in HPV8 E6 expressing HaCaT cells (see Fig 4D and 4E, S3A Fig). The hsa-miR-203 mimic reduced p63 protein and ΔNp63α mRNA levels in the controls and reverted p63 almost to basal levels in HPV8 E6 expressing HaCaT cells (see Fig 4G and 4H, S3B Fig). Notably, the ability of HPV8 E6-expressing cells to close the gap in the scratch assay was significantly impaired after p63 knock-down (Fig 3D) or miR-203 mimic transfection (Fig 3E).

We then studied whether the miR-203 pathway is involved in HPV8 E6-mediated suppression of differentiation. Organotypic cultures of HPV8 E6 expressing HaCaT cells further revealed a dramatic reduction of the differentiation marker involucrin compared to controls (Fig 4A and S3C Fig). In monolayer cultures of HPV8 E6 expressing HaCaT cells treated with 1.2 mM Ca^2+^ for 72 h involucrin induction was potently suppressed at protein and mRNA levels (Fig 4B and 4C). Notably, p63-specific siRNA led to a significant up-regulation of involucrin expression in the control cells and reverted involucrin protein and mRNA expression in the HPV8 E6 positive cells to basal levels (Fig 4D and 4F, S3D Fig). Also miR-203 mimic transfection led to a significant up-regulation of involucrin expression in E6 expressing HaCaT cells (Fig 4G and 4I, S3E Fig). These data provided evidence that keratinocyte reprogramming by HPV8 E6 involves the miR-203/p63 pathway.

### C/EBPα is a direct transcriptional regulator of miR-203

**Fig 5:**
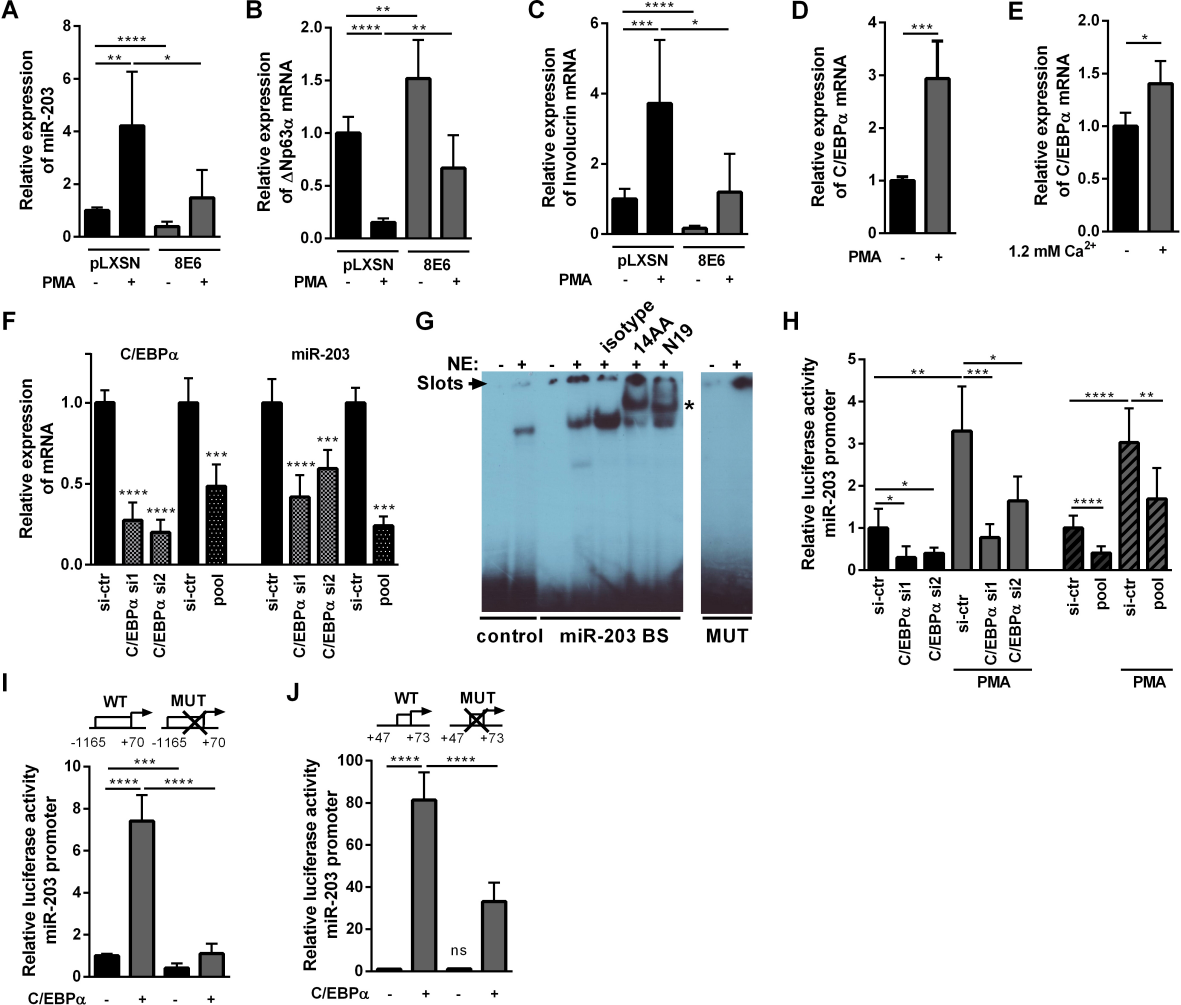
C/EBPα is a direct transcriptional regulator of miR-203. **(A-C)** NHK stably expressing HPV8 E6 or corresponding pLXSN control cells were stimulated with 50 ng/ml PMA for 24 h. **(A)** miR-203 levels were measured in qRT-PCR and normalized to RNU6B by the 2^ΔΔCt^ method. **(B)** ΔNp63α and **(C)** involucrin mRNA expression levels were measured by qRT-PCR and normalized to RPL13A. NHK pLXSN control cells were analyzed for C/EBPα mRNA expression **(D)** 24 h after 50 ng/ml PMA or **(E)** 72 h after 1.2 mM Ca^2+^ stimulation. **(F)** NHK were transfected with 10 nM siRNA (pool or two single siRNAs) against C/EBPα (or si-control). C/EBPα mRNA or miR-203 expression levels were determined by qRT-PCR. **(G)** ^32^P-labeled oligonucleotides containing a C/EBP binding site from the IL-6 gene (control) or a predicted binding site within the miR-203 hairpin sequence (miR-203 BS, nt +57 to +63) or its mutated form (MUT) were incubated with 5 μg (control) or 20 μg (miR-203 BS, MUT) nuclear extracts (NE) from C33a cells transfected with pcDNA3.1-C/EBPα expression vector and analyzed by EMSA. For supershift analysis, nuclear extracts were pre-incubated with 2 μg of two different antibodies against C/EBPα (clone 14AA and N19) or respective isotype control. Asterisk indicates supershifted bands. Gel slots are indicated by an arrow. **(H)** NHK were transfected with 10 nM siRNA (pool or two single siRNAs) directed against C/EBPα (or si-control) 24 h before transfection of the miR-203 promoter reporter construct comprising the miR-203 regulatory region (-1165 nt to +70). Cells were stimulated with 50 ng/ml PMA for 24 h. Luciferase activity was measured and normalized to protein concentrations of the respective luciferase extracts. **(I, J)** HaCaT cells were co-transfected with 0 or 100 ng C/EBPα expression vector and wildtype (WT) miR-203 reporter (in I from -1165 nt to +70 nt; in J from +47 nt to +73 nt) or respective mutated miR-203 reporter constructs (MUT, comprising the mutated C/EBPα binding site within the miR-203 hairpin sequence) and luciferase activity was determined. Mean values ± SD from *n* ≥ 3 experiments performed in duplicates are shown. (*p<0.05, **p<0.01, ***p<0.001, ****p<0.0001).

The impact of HPV8 E6 on mir-203, p63 and involucrin expression was also investigated in NHK after stimulation with phorbol 12-myristate 13-acetate (PMA), which induces differentiation in cultured normal human keratinocytes [34, 40]. Expression of HPV8 E6 significantly counteracted PMA-induced miR-203 and involucrin up-regulation as well as PMA-mediated ΔNp63α suppression in NHK (Fig 5A–5C).

Notably both, PMA and Ca^2+^, induce the transcription factor C/EBPα (Fig 5D and 5E) and its DNA-binding activity in murine or human keratinocytes [34, 41, 42]. C/EBPα is a potent inducer of keratinocyte differentiation [34, 35]. It has been characterized as a tumor suppressor in multiple tissues including skin [43], and inactivation of C/EBPα in mice strongly supports UV-induced skin carcinogenesis [44] qualifying C/EBPα as an interesting potential target of HPV8 E6. Importantly, when we knocked-down C/EBPα in NHK, this did not only reduce C/EBPα mRNA but also potently suppressed endogenous miR-203 expression (Fig 5F) indicating that C/EBPα plays a role in miR-203 regulation. C/EBPα knock-down efficiencies were 52–80% as quantified by RT-PCR (S3 Table). C/EBPα protein knock-down was confirmed by IHC (S4A Fig).

We further explored a potential role of C/EBPα as a direct transcriptional regulator of miR-203. In silico analysis of the *miR-203* gene revealed a C/EBPα binding site (GCGCAAT, nt +57 to +63) within the miR-203 hairpin sequence. EMSAs with nuclear extracts from C/EBPα overexpressing cells showed binding to a well-known C/EBP-binding site [45] as well as to the specific C/EBP-binding site within the miR-203 hairpin sequence (Fig 5G). Two different antibodies directed against C/EBPα (clone 14AA and N19) resulted in a supershift. C/EBPα did not bind to oligonucleotides comprising a mutated binding site (GC**T**C**G**A**G**). These experiments demonstrated for the first time binding of the transcription factor C/EBPα to a specific binding site within the *miR-203* gene.

We then generated a miR-203-reporter construct (-1165 nt to +70 nt) comprising the miR-203 hairpin sequence and investigated the functional impact of C/EBPα. PMA strongly stimulated this miR-203-reporter construct in NHK (Fig 5H), while knock-down of C/EBPα significantly reduced basal as well as PMA-stimulated reporter activity. Co-transfection of a C/EBPα-encoding expression plasmid led to a more than 7-fold induction of reporter activity with wildtype C/EBPα-BS, which was significantly reduced when this site was mutated to the C/EBPα-binding deficient form (Fig 5I). Similar results were obtained when drastically shortened forms of a miR-203 reporter construct (+47 nt to +73 nt) were used comprising either the wildtype or the mutated C/EBPα-BS but largely omitting other regulatory regions within the miR-203 gene, (Fig 5J). These results demonstrated a direct role of C/EBPα for miR-203 transcription.

### HPV8 E6 suppresses C/EBPα expression

**Fig 6:**
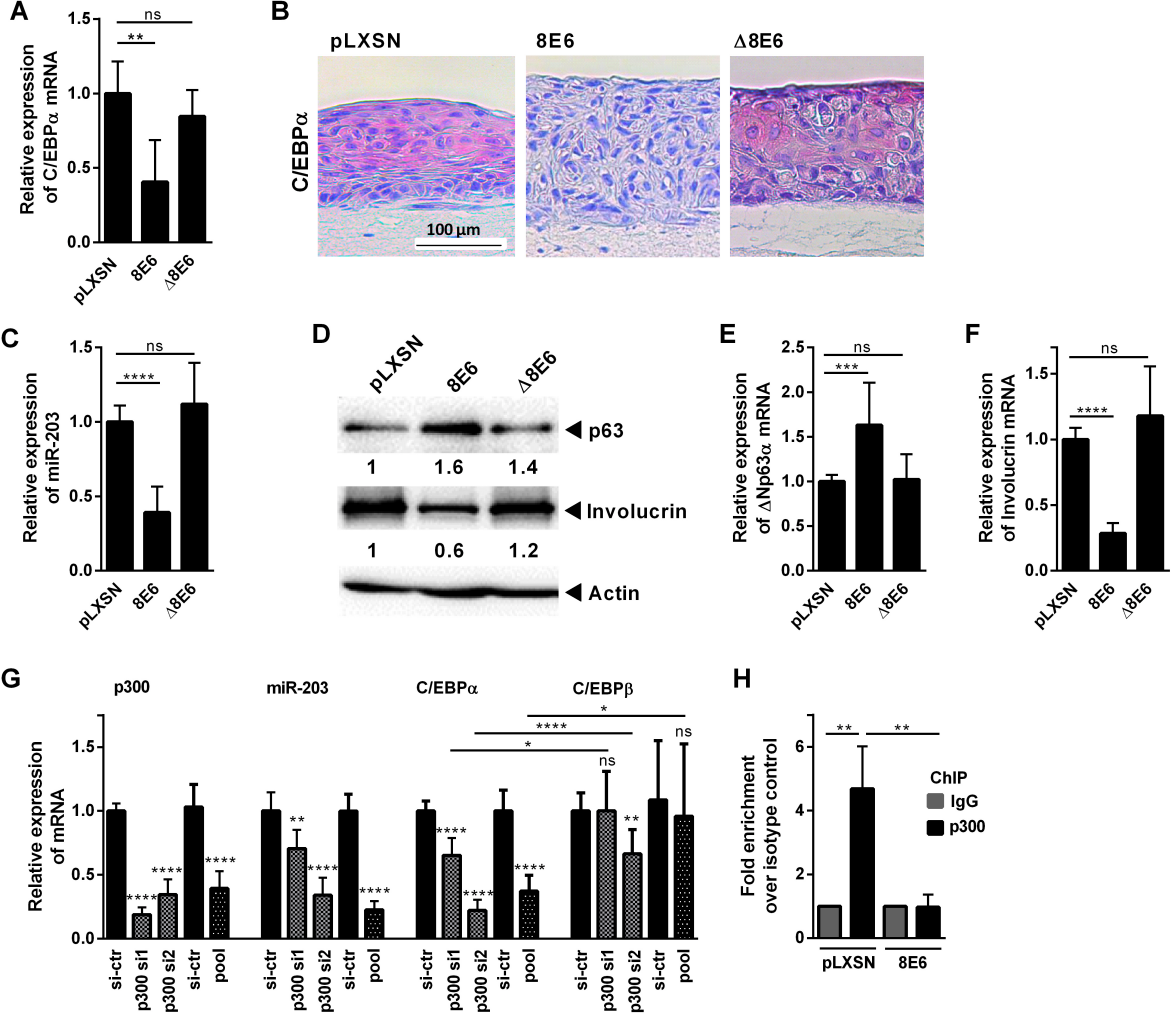
p300 regulates C/EBPα and miR-203 expression. **(A)** NHK stably expressing HPV8 E6, mutant Δ8E6 (Δaa132-136) deficient in p300-binding, or pLXSN control cells were analyzed for C/EBPα expression by qRT-PCR. **(B)** C/EBPα protein expression was determined by IHC in HPV8 E6, Δ8E6 expressing HaCaT or control organotypic cultures (red). **(C)** In retrovirally infected NHK cells miR-203 expression was measured by qRT-PCR, **(D)** p63 and involucrin protein expression was determined by Western blot in relation to actin (shown is one representative blot out of three). **(E)** ΔNp63α and **(F)** involucrin mRNA expression levels were measured by qRT-PCR normalized to RPL13A. **(G)** NHK were transfected with 10 nM p300-specific siRNA (pool or two single siRNAs versus si-control), harvested 48 h later and expression of p300 mRNA, miR-203, C/EBPα and C/EBPβ mRNA were quantified by qRT-PCR as described. Mean values ± SD from *n* ≥ 3 experiments performed in duplicate are shown. **(H)** HPV8 E6 expressing HaCaT or pLXSN control cells were subjected to chromatin immunoprecipitation. Protein-genomic DNA complexes were precipitated with anti-p300 antibody. DNA was isolated, amplified by real-time PCR with primers specific for the nt -1533 to -1398 regulatory region of the *CEBPΑ* gene. The amount of target DNA precipitated with the isotype control antibody was set at 1. The mean values ± SD from 3 independent experiments are presented. (ns: not significant, *p<0.05, **p<0.01, ***p<0.001, ****p<0.0001).

Since our data revealed C/EBPα as a novel inducer of miR-203 expression we were interested whether HPV8 E6 might regulate this important transcription factor. Notably, HPV8 E6 expressing keratinocyes showed strongly reduced C/EBPα mRNA levels (Fig 6A) and this was confirmed at the protein level in organotypic cultures (Fig 6B). In epithelia formed by HPV8 E6 expressing HaCaT cells, C/EBPα expression was almost undetectable. Moreover, C/EBPα induction was potently inhibited in PMA- or Ca^2+^-stimulated HPV8 E6 expressing keratinocytes (S5A and S5B Fig). HPV8 E6-mediated suppression was highly specific for C/EBPα, since C/EBPβ, another transcription factor of the C/EBP family, was not reduced in HPV8 E6 expressing keratinocytes (S5C Fig).

### C/EBPα expression depends on p300

To get a hint on HPV8 E6-mediated regulation of C/EBPα, we investigated a HPV8 E6 mutant lacking amino acids 132–136 (variously named “Δ8E6”) deficient in p300 binding [18, 46]. We generated this mutant and expressed it by retroviral gene transfer in NHK and HaCaT cells. When compared to HPV8 wildtype-E6, the Δ8E6 mutant was unable to suppress miR-203 and involucrin and to induce p63 at mRNA and protein levels in NHK (Fig 6C–6F). Notably, the Δ8E6 mutant was also unable to suppress C/EBPα mRNA and protein expression (Fig 6A and 6B).

To further explore a role of endogenous p300 for miR-203 expression, we knocked-down p300 in NHK as confirmed by Western blot analysis (S4B Fig). p300 knock-down efficiencies were between 60 and 81% as quantified by RT-PCR (S3 Table). This potently suppressed miR-203 (Fig 6G). Surprisingly, p300 knock-down also significantly reduced the expression of the transcription factor C/EBPα (Fig 6G), while the related transcription factor C/EBPβ, which was determined in the same extracts, was significantly less affected by all p300-specific siRNAs. These data indicated that p300 is involved in *CEBPΑ* gene expression.

p300 is a histone acetyltransferase and histone acetylation in the regulatory region of the *CEBPΑ* gene can be linked to C/EBPα expression levels [47]. We therefore assessed p300 binding to C/EBPα chromatin in HPV8 E6 expressing HaCaT and pLXSN control cells in a regulatory region previously shown to be susceptible to histone acetylation (nt -1533 to -1398) [47]. As shown by chromatin immunoprecipitation (ChIP), p300 bound to this regulatory region in pLXSN control cells and this binding was significantly reduced in the HPV8 E6 expressing cells (Fig 6H). Together these data provided evidence that p300 binds to the regulatory region of C/EBPα, a novel inducer of miR-203, and that HPV8 E6 interferes with this binding activity.

### C/EBPα and miR-203 are both down-regulated in the lesional epidermis of EV-patients

**Fig 7:**
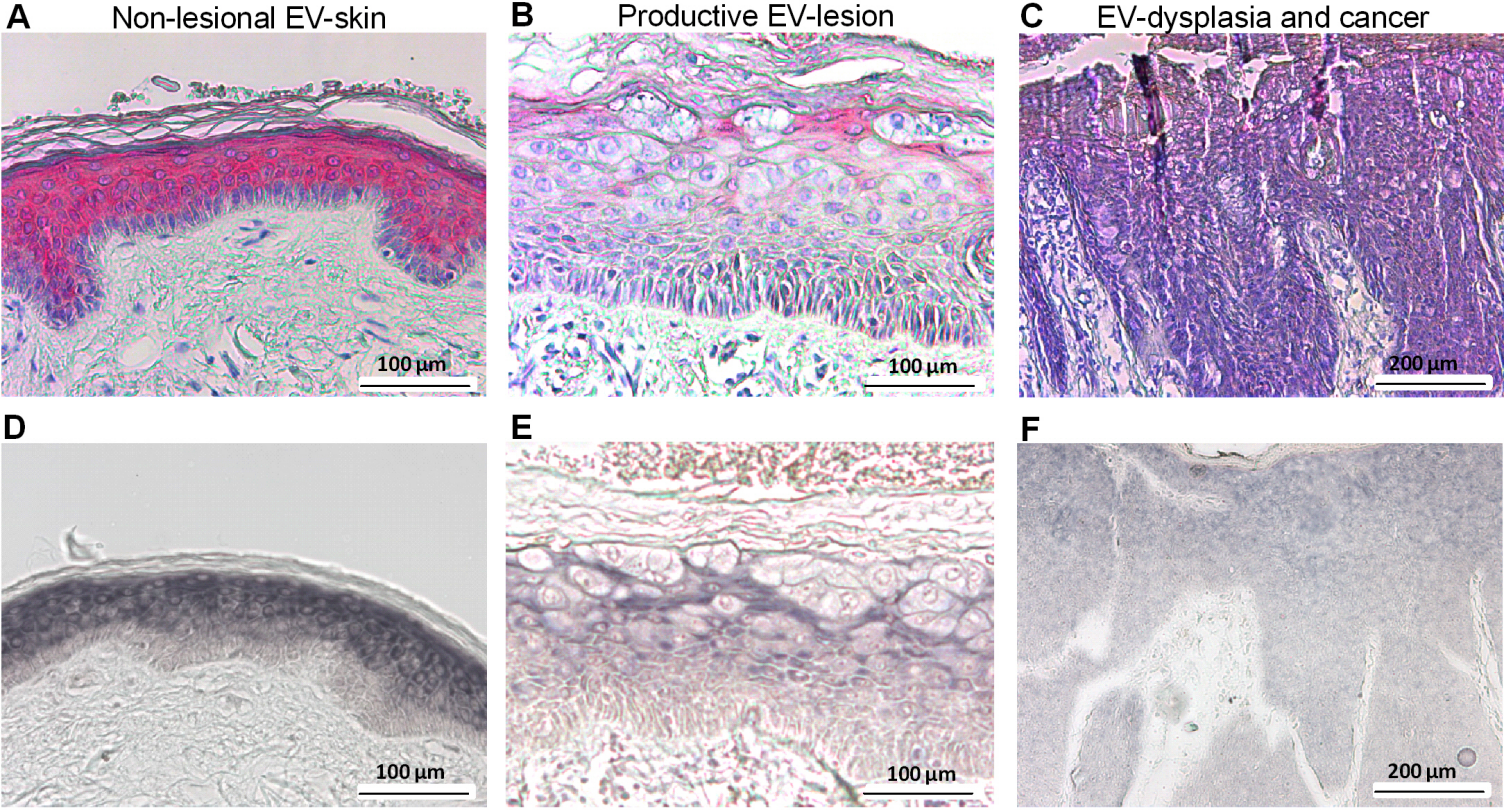
C/EBPα and miR-203 expression in non-lesional and HPV8-positive lesional skin of Epidermodysplasia verruciformis patients. Sections of non-lesional skin **(A, D)** and HPV8-positive lesional skin **(B, C, E, F)** from EV-patients were stained in red using antibodies against C/EBPα **(A-C)** and in blue by in situ hybridization with a probe against miR-203 **(D-F)**. Nuclear counterstaining was performed with hematoxylin solution (A-C).

In order to corroborate our findings in vivo we stained skin sections of EV-patients for C/EBPα and compared the respective expression patterns to miR-203 by in situ hybridization. In non-lesional skin C/EBPα was restricted to suprabasal, differentiated layers of the epidermis (Fig 7A). This pattern matched well with miR-203 expression pattern in the same biopsy (Fig 7D). HPV8-positive EV-lesions showing viral cytopathic effects displayed strongly reduced expression of C/EBPα and miR-203 (Fig 7B and 7E) and in highly dysplastic or invasive lesions expression of both factors was almost undetectable (Fig 7C and 7F). In contrast, the miR-203 target p63 displayed complementary expression patterns (Fig 1A and 1B, S6 Fig) corresponding well to the in vitro data.

## Discussion

**Fig 8:**
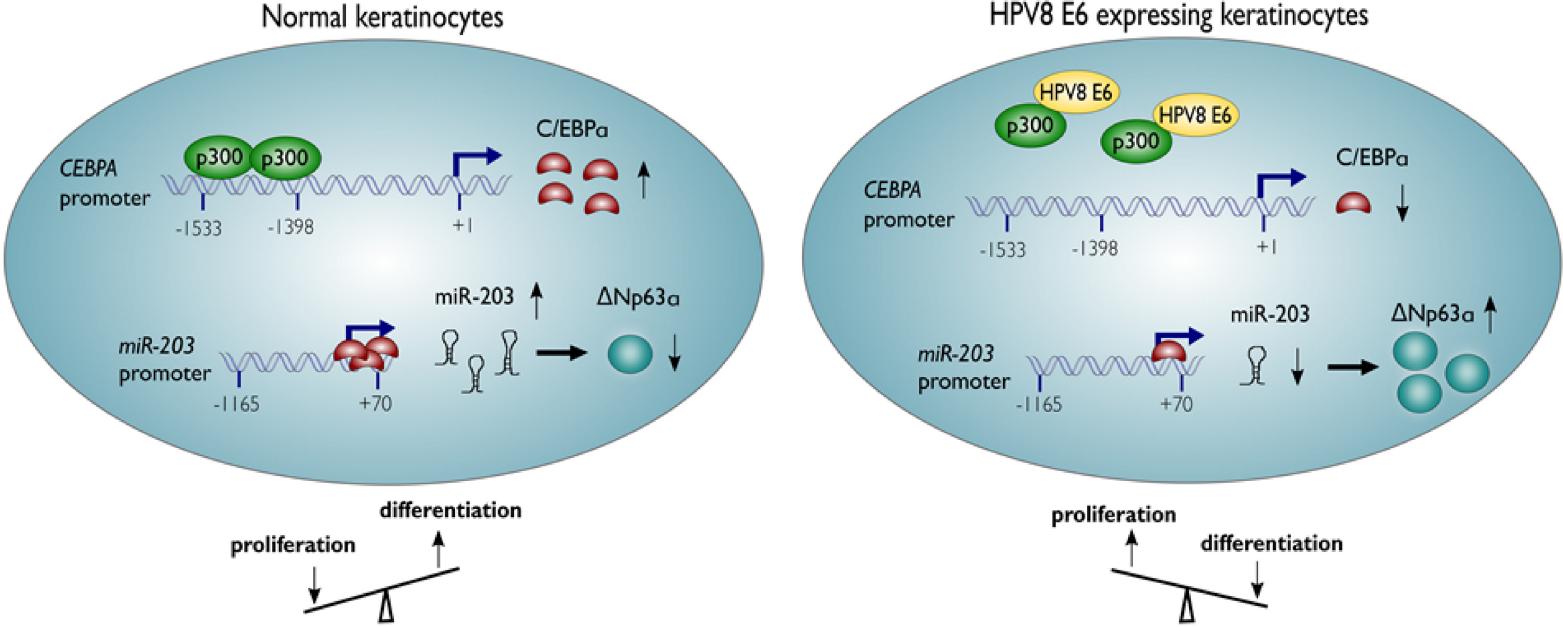
Schematic presentation. A novel p300/C/EBPα/miR-203-dependent pathway by which the cutaneous HPV8 E6 protein may expand p63-positive keratinocytes and disturb epidermal homeostasis.

Our study for the first time documents that miR-203, a key regulator of epidermal proliferation and differentiation, is potently down-regulated in HPV8-positive EV-lesions. We have identified C/EBPα, a differentiation-inducing transcription factor as a novel inducer of miR-203 and the HPV8 E6 protein as a potent suppressor of C/EBPα and miR-203 expression. C/EBPα down-regulation involves p300, a well-described target of the HPV8 E6 protein. HPV8 E7, by contrast, had a less strong effect on miR-203 expression but was able to enhance the impact of E6. In situ stainings confirmed congruent suprabasal expression patterns of C/EBPα and miR-203 in non-lesional skin of EV-patients and strong down-regulation of both factors in HPV8-positive EV-lesions in vivo, supporting our in vitro data. Key findings of this study are summarized in Fig 8.

MiR-203 is a suppressor of self-renewal in skin [22]. It represses keratinocyte ‘stemness’ by targeting ΔNp63α in skin keratinocytes [21], induces cell-cycle exit [22] and promotes epidermal differentiation [20]. Moreover, miR-203 targets other cellular factors including c-myc [48], another important regulator of proliferation, suppressor of cytokine signaling 3 (SOCS3) [49] or IL-8 [50] and it cooperates with other miRNAs to suppress the B lymphoma Mo-MLV insertion region 1 homolog (BMI1), which is involved in self-renewal [51]. This suggests that miR-203 and further targets aside from ΔNp63α might play a broader role in E6-mediated reprogramming of the host cell during cutaneous beta-HPV infection. In a preliminary gene expression analysis of primary human keratinocytes expressing the HPV8 E6 oncoprotein, we did, however, not observe up-regulation of c-myc but a less than 2-fold decrease. IL-8 even appears to be suppressed by HPV8 E6 [52] pointing to the regulation of IL-8 by pathways other than miR-203 in HPV8 E6-positive keratinocytes.

While miR-203 function has been intensively investigated, little is known about the regulation of miR-203 expression in human keratinocytes. Our data demonstrate that C/EBPα can bind to a predicted binding site within the *miR-203* gene [22] thereby stimulating miR-203 transcription. Lack of miR-203 promoter activation by C/EBPα using constructs comprising a mutated C/EBPα binding site confirmed a direct effect of C/EBPα on miR-203 transcription. The finding that C/EBPα, a member of the CCAAT/enhancer-binding protein family, is involved in miR-203 regulation is particularly interesting. Both, C/EBPα and β, are expressed in a differentiation-dependent manner in the epidermis and play an essential role for interfollicular keratinocyte proliferation arrest, commitment and differentiation [5, 35, 42]. We had previously shown that the HPV8 E7 protein specifically interacts with C/EBPβ via the C-terminal FQELL sequence thereby interfering with its transcriptional activity [5]. Here, we demonstrate that HPV8 E6 potently suppresses C/EBPα at transcriptional and protein levels as demonstrated in monolayer and organotypic cultures. These data were strongly supported by the observation that C/EBPα is dramatically down-regulated in EV-lesions in vivo. Notably, C/EBPβ mRNA levels were rather unaffected by HPV8 E6. Thus, the HPV8 E6 and E7 oncoproteins target both C/EBP factors, albeit via different mechanisms.

MiR-203 is also suppressed by mucosal HR-HPV, and this is necessary to maintain genome amplification in keratinocytes under differentiating conditions [30]. HPV16 E6 and HPV16/31 E7 were shown to down-regulate miR-203 expression via distinct mechanisms [30, 31]. HPV16 E6 prevents miR-203 expression by targeting p53. How p53 affects miR-203 is currently unclear, since p53 did not directly bind to predicted binding sites within the miR-203 promoter [31]. Notably, in our study HPV8 E6 efficiently suppressed miR-203 also in the p53-mutated cell line HaCaT [37]. Mucosal HR-HPV E7 interfered with differentiation-induced miR-203 involving the PMA-inducible MAPK/PKC-pathway [30]. PMA activates several differentiation-inducing pathways including transcription factors of the AP-1- and C/EBP-families, which are both involved in PMA-induced miR-203 regulation ([23] and this study). Interestingly, C/EBPα also seems to be a target of the HPV16 E7 protein. In murine fibroblasts HPV16 E7 was reported to interfere with C/EBPα-mediated proliferation arrest, while ectopic overexpression of HPV16 E7 in these cells had no impact on C/EBPα-induced adipogenic differentiation [53]. It remains to be determined whether or not mucosal HPV oncoproteins can regulate endogenous C/EBPα in keratinocytes, the natural host cells of HPV, as has been demonstrated for the HPV8 E6 protein in this study.

Our study provides further mechanistic insight into the transcriptional regulation of C/EBPα. The histone acetyltransferase p300 is a well-known transcriptional co-activator of C/EBPα [54] and a well-described target of cutaneous papillomaviral E6 proteins [18, 19, 46, 55]. In the CRPV-model, the E6-p300 interaction was found to be crucial for tumorigenesis [19]. Interestingly, our knock-down experiments provided evidence that endogenous p300 is involved in the regulation of C/EBPα mRNA expression in keratinocytes. The HPV8 Δaa132-136-E6 deletion-mutant, which is unable to bind to p300 [18, 46], neither conferred down-regulation of C/EBPα in organotypic cultures nor of its transcriptional target miR-203. The question has been raised whether this deletion-mutant of HPV8 E6 could be defective for more than p300 binding [17]. Of note, in the same series of experiments, we observed that HPV8 E6 also affects expression of the AP-1 transcription factor JunB. In contrast to C/EBPα, however, JunB was efficiently down-regulated by Δ8E6, and this suppression did not significantly differ from HPV8 E6-wildtype mediated JunB suppression (S7A Fig). This demonstrated that the HPV8 Δaa132-136-E6 deletion-mutant retains some functions observed for wildtype HPV8 E6 indicating that it is not generally functionally defective. Moreover, p300 knock-down did not suppress JunB expression (S7B Fig) further underlining the specificity of p300 in C/EBPα regulation. The regulatory region of the *CEBPΑ* gene was previously shown to be sensitive to histone acetylation and the acetylation status correlated with C/EBPα expression levels [47]. Importantly, in HPV8 E6 expressing keratinocytes, which display strongly reduced C/EBPα expression, we found significantly less p300 binding to this C/EBPα regulatory region by ChIP. This indicated that p300 might act directly on the *CEBPΑ* gene. However, since p300 can affect the expression of numerous genes and has broad effects on acetylation of many proteins, it is reasonable to assume that indirect p300 effects could also contribute to the regulation of *CEBPΑ* gene expression.

In our experiments HPV8 E6 up-regulated the ‘stemness’ factor ΔNp63α [26] as shown by isoform-specific RT-PCR in primary human keratinocytes, induced proliferation or migration and suppressed differentiation. This involved suppression of miR-203. Cell cycle analysis revealed a reduction of the HPV8 E8 expressing cells in G1-phase and a significant increase of cells in the S-phase. These data are in line with previous observations demonstrating an impact of genus beta-HPV oncoproteins on keratinocyte proliferation and differentiation [15, 18, 56, 57]. ΔNp63α may in turn increase genus beta-HPV promoter activity as demonstrated for HPV20 [58].

E6 proteins encoded by genus beta-HPVs can also associate with Mastermind-like 1 (MAML1), a transcriptional co-activator and mediator of canonical Notch signaling. E6 thereby interferes with Notch signaling and delays Notch-dependent differentiation [14–17, 59]. While Notch1 suppresses p63 expression [60], this appears to be independent of canonical Notch signaling [61]. In fact, experimental data in a different study indicated that “p63 expression in differentiating” HPV8 E6-positive keratinocytes “may not be strictly NOTCH-dependent” [15]. However, MAML1 cooperates with p300 in a transcriptional activator complex and potentiates p300 autoacetylation [62]. Thus, there might be a link to the pathway described in this study. In fact, our own preliminary data show that knock-down of MAML1 by siRNA contributes to the down-regulation of C/EBPα (S7C Fig) indicating a cross-talk between Notch- and C/EBPα-signaling, which will be interesting to study in the future.

In summary, we have identified a novel p300/C/EBPα/miR-203/p63 pathway important for epidermal homeostasis. Our study provides evidence that this C/EBPα-dependent pathway, which fundamentally reprograms keratinocyte function, is counteracted by HPV8 E6.

C/EBPα has a substantial role not only for the regulation of keratinocyte proliferation and differentiation [34, 35] but it is also implicated in their DNA-damage G1 checkpoint response [63]. Notably, C/EBPα can function as a tumor suppressor [43, 64]. The *CEBPA* gene is mutated in acute myeloid leukemia [65]. Moreover, C/EBPα expression is down-regulated in various tumor entities including cervical, head and neck as well as skin cancer [43, 66, 67] and re-introduction of C/EBPα in carcinoma cells can inhibit their growth [68]. Strikingly, loss of C/EBPα also confers susceptibility to UV-induced skin carcinogenesis [44].

Thus, it is tempting to speculate that HPV8 E6 may contribute to the oncogenic functions of HPV8 in the skin of EV-patients and potentially also in immunosuppressed patients where the virus is expressed, via down-regulation of C/EBPα.

## Materials and methods

### Ethics statement

The study was approved by the Saarland University at the Saarland Ärztekammer and the Bioethics Committee at the Medical University of Warsaw, Poland and conducted according to the principles expressed in the Declaration of Helsinki. The data were analyzed anonymously.

### Plasmid constructs

pcDNA3.1-C/EBPα, pLXSN-8E6, pLXSN-8E7, pLXSN 8E6/E7, pCMV-eGFP were previously described [5, 69]. The pLXSN-Δ8E6 binding mutant was generated by site-directed mutagenesis according to [46].

The hsa-miR-203 promoter (-1165 nt to +70 nt; nomenclature according to [51] was amplified from human DNA using 5’-*ACGCGT*TGCTGCCCAACCCCATAC-3’ and 5’-*CTCGAG*CTCCCCTGGATTGGTCGC-3’ (restriction enzyme sequences in italics) and cloned upstream a firefly luciferase into pGL3-Basic vector (Promega, Madison, USA). Mutagenesis of the C/EBPα BS was performed using Q5 site-directed mutagenesis kit (New England BioLabs) according to the manufacturer`s protocol using 5’-GTTCTGTAGC**T**C**G**A**G**TGTGAAATGTTTAG-3’ and 5’-TGTTGAACTGTTAAGAACC-3’ oligonucleotides. The short hsa-miR-203 promoter (+47 nt to +73 nt) was generated by direct cloning of annealed oligonucleotides into pGL3Basic vector (wildtype: 5’-*CGCGT*CAGTTCTGTAGCGCAATTGTGAAATGT*C*-3’ and 5’-*TCGAG*ACATTTCACAATTGCGCTACAGAACTG*A*-3’; mutated: 5’-*CGCGT*CAGTTCTGTAGC**T**C**G**A**G**TGTGAAATGT*C*-3’ and 5’-*TCGAG*ACATTTCACA**C**T**C**G**A**GCTACAGAACTG*A*-3’). Restriction sites are indicated in italics, the C/EBPα binding site is underlined, mutations are in bold. All constructs were verified by DNA sequencing.

### Cell culture, retroviral infection and organotypic cultures

NHK were isolated from foreskin tissue (Saarland University Medial Center) and cultured in supplemented KBM-Gold medium (Lonza, Basel, Switzerland). NHK and the spontaneously immortalized keratinocyte cell line HaCaT (a gift from Dr. P. Boukamp, German Cancer Research Center, Heidelberg, Germany) [37] were retrovirally engineered to express HPV8E6, E7 or E6/E7 using the pLXSN vector system (BD Biosciences, Heidelberg, Germany) as described [5]. HaCaT and the HPV-negative cervical carcinoma cell line C33A (HTB-31; American Type Culture Collection, Manassas, VA) were cultured in supplemented DMEM [70]. For differentiation-inducing experiments 6x10^5^ or 1x10^5^ cells were seeded onto 10 cm dishes or 6-well plates, respectively, treated with 1.2 mM CaCl_2_ (Sigma Aldrich, Steinheim, Germany) and harvested after 72 h. To generate organotypic cultures, 3x10^5^ foreskin fibroblasts, passage 3–5, were seeded in 4 mg/ml rat-tail collagen (as described in [71]) on a collagen-fleece (MedSkin Solutions, Billerbeck, Germany) in 24-well plates and cultured in DMEM medium. Next day, 6x10^5^ HaCaT-pLXSN or 8E6 expressing HaCaT were seeded onto the collagen-fibroblasts in DMEM containing 25% Ham's F-12 medium with supplements as described in [69]. 100 U/ml penicillin/streptomycin was used as antibiotic. 24 h later, cultures were transferred on a metal grid in 6-well plates to culture them at the liquid-air interface. Two weeks later the cultures were fixed in 4% formaldehyde and embedded in paraffin. To verify C/EBPα knock-down at the protein level, HaCaT cells were transfected with 10 nM siRNA or control siRNA 16 h before seeding on collagen/fibroblasts and cultured for 6 d at the liquid-air interface.

### Transient transfection and luciferase assay

2.5x10^5^ cells were seeded into 6-well plates and transfected the next day with 10 nM siRNA directed against p63, p300, C/EBPα, 20 nM hsa-miR-203 mimic or control siRNA (S1 Table) and 5 μl Lipofectamine RNAiMax reagent (Invitrogen, Darmstadt, Germany). The cells were harvested for RNA or protein after 48 h. For luciferase assays 1x10^5^ cells were seeded into 12-well plates and transfected with 200 ng pGL3Basic-miR-203 promoter constructs, 100 ng pEGFP-C1, 100 ng pcDNA3.1-C/EBPα and 2 μl Lipofectamine 2000 (Invitrogen) or 2.4 μl Transfast (Promega) for HaCaT or NHK, respectively as described in [72]. The total amount of DNA was adjusted to 0.8 μg with the respective empty vectors. 48 h post transfection cells were checked for transfection efficiencies by FACS analysis and lysed with luciferase extraction buffer containing 0.1 M potassium phosphate buffer (pH 7.5) and 0.5% IGEPAL-630. Luciferase activity was determined with a Victor II luminometer (Perkin-Elmer LAS) and normalized to protein concentrations and transfection efficiencies in the respective extracts. In some experiments cells were treated with 50 ng/ml PMA (Sigma-Aldrich) 24 h after transfection for additional 24 h. In some experiments, cells were transfected with 10 nM C/EBPα siRNA or control siRNA 24 h prior to transfection with the promoter construct.

### Quantitative real-time PCR

cDNA was generated from 1 μg RNA with SuperscriptII (Invitrogen). qRT-PCR was performed with the LightCycler 480II (Roche, Mannheim, Germany) using the Universal Probe System (Roche). Expression levels were normalized to ribosomal protein L13A (RPL13A), which is a well-proven housekeeping gene in keratinocytes [73], or beta-actin as described [74]. Intron-spanning oligonucleotides were designed with the Universal Probe Library (UPL) software (Roche) (S2 Table).

miRNA was transcribed using TaqMan MicroRNA RT Kit (Life technologies) with oligonucleotides for hsa-miR-203 and hsa-RNU6B in each reaction. miRNA-cDNA detection was based on TaqMan MicroRNA Assays (Life Technologies). The amount was calculated by the 2^–[Δ][Δ]Ct^ method normalizing to RNU6B.

### Proliferation, cell cycle and scratch assays

The BrdU Assay was performed according to the manufacturer`s protocol (Roche). 1.5x10^4^ cells/96-well plate were seeded in triplicates in supplemented KBM-Gold medium. After 20 h, cells were serum-starved. 24 h later the medium was replaced by supplemented KBM-Gold with 10 μM BrdU. The incorporation of BrdU was detected after 17 h by anti-BrdU-peroxidase labeled antibody. Enzymatic reaction with TMB (tetramethyl-benzidine) was measured after stopping the reaction with 2 N H_2_SO_4_ in the Victor II plate reader at 420 nm. For DNA cell cycle analysis, HaCaT pLXSN and HPV8 E6 expressing cells seeded at a cell density of 3x10^5^ in 6-well plates were fixed 4 d later in acetone/methanol for 10 min at -20°C. Cells were treated for 1 h with 1 mg/ml RNase and stained with 5 μg PI per 100 μl cells for 30 min on ice to determine the DNA content by flow cytometry at 488 nm (FACSCanto II, BD Biosciences). The single cell population was gated and cell counts were measured in the PI histogram plot to calculate G0/G1-, S- and G2/M-phases by FACSDiva software 8.0.1 (BD Biosciences). For scratch assay HaCaT monolayers were scratched in 4 cm-dishes. The cells were analyzed at time points 0, 24 and 48 h with Leica DMI600B microscope and Leica application suite software (LAS) V3.06. Cells transfected with p63 siRNA or hsa-miR-203 were scratched 24 h after transfection.

### Immunohistochemical staining and miR-203 in situ hybridization

Anonymized lesional and non-lesional formalin fixed paraffin embedded (FFPE) skin specimens from EV-patients were obtained from the Department of Dermatology, Medical University of Warsaw, Poland. The presence of HPV8 was verified by qRT-PCR as described in [75]. Antigen retrieval was performed by heating the sections in 1 mM citrate buffer pH 7.0 at 95°C for 10 min and endogenous peroxidase activity was blocked with 3% H_2_O_2_/TBS for 10 min (for DAB). Rabbit anti-C/EBPα antibody 14AAx (1:10000, Santa Cruz), mouse anti-involucrin SY5 (1:80000, Sigma-Aldrich) and mouse anti-p63 antibody 4A4 (1:400, Santa Cruz) were used. FFPE sections were incubated with the indicated antibodies overnight (C/EBPα) or for 1 h (involucrin, p63) followed by HRP-conjugated secondary anti-mouse or AP-conjugated anti-rabbit antibody incubation and developed with DAB or AP-substrates. After counterstaining with hematoxylin the slides were covered with Vectamount and evaluated with Leica LAS.

For microRNA in situ hybridization 4 μm sections of FFPE EV-biopsies were dried for 1 h at room temperature and overnight at 37°C. After deparaffinization with xylol and rehydration, the slides were incubated in 15 μg/ml proteinase K for 10 min at 37°C, followed by dehydration with increasing ethanol concentrations. 5 nM hsa-miR-203 detection probe (cat. 88079–15, Exiqon) was denaturated for 4 min at 90°C and the slides were incubated for 1 h/42°C with 25 μl of hybridization mix. After stringent washing with SSC buffers in decreasing concentrations, the slides were blocked (DIG wash and block buffer, Roche Applied Science) and incubated with 50 μl anti-DIG-AP antibody (1:800, Roche) for 1 h in a humid chamber followed by a three time washing step in PBS/1% Tween-20. The slides were incubated for 1 h in 200 μl developing substrate (NBT/BCIP, Roche Applied Science), washed with KTBT buffer (50 nM Tris-HCl, 150 mM NaCl, 10 mM KCl) and dehydrated. After covering with Entellan, the FFPE-sections were analyzed by Leica LAS. The Immunoreactive Score (IRS) according to Remmele & Stegner [76, 77] was used to quantify C/EBPα protein expression in organotypic cultures in C/EBPα siRNA experiments.

### Western blot

Cells were lysed in 2x buffer containing 6.3% SDS, 10% glycerine, 9.5% mercaptopropandiole, 4% uppergel stock containing 0.5 M Tris and 0.4% SDS, pH 6.8. 15 μg protein extract were analyzed by Western blot as described in [77] using mouse anti-involucrin SY5 (1:5000, Sigma-Aldrich), mouse anti-p63 4A4 (1:5000, Santa Cruz), mouse anti-p300 RW128 [78] (1:1000, Millipore) and mouse anti-β-actin A-5441 (1:5000, Sigma-Aldrich) antibodies. The p300 antibody is non-cross reactive to the homologue CBP (as indicated by epitope amino acid sequence).

### Northern blot

Northern blot was performed as previously described [79] with little modifications. 15 μg of total RNA, isolated with miRNeasy Kit (Qiagen, Hilden, Germany), were resolved on 12% urea-acrylamide-bisacryamide gel (National Diagnostics, Atlanta, USA). RNA was transferred on nylon membrane by semi-dry blotting for 30 min at 15 V. After 1.5 h of chemical crosslinking (0.13 M methylimidazole, 0.16 M EDC hydrochloride) at 55°C the membrane was incubated overnight with the ^32^P-dUTP labeled probe (miRVana probe construction Kit, Ambion, Life technologies). The following antisense probe was used to detect miR-203: 5’-GTGAAATGTTTAGGACCACTAG-3’. The signal was detected with PhosphoImager (Molecular Dynamics, Sunnyvale, USA).

### Electromobility shift assays (EMSA)

A putative C/EBPα binding site was identified using the MatInspector program from Genomatix V8.4 and ALGGEN PROMO [80, 81]. A dsDNA oligonucleotide comprising this binding site (C/EBPα BS: 5’-GTTCTGTAGCGCAATTGTGAAATGTT-3’ or MUT 5’-GTTCTGTAGC**T**C**G**A**G**TGTGAAATGTT-3’) was labeled with (γ-^32^P)-ATP (Perkin-Elmer) using T4 polynucleotide kinase (New England Biolabs, Ipswich, MA). Nuclear extracts from C33A cells transfected with pCDNA3.1-C/EBPα were prepared as described [82], incubated in EMSA buffer [5] at room temperature for 15 min and analyzed as described in [5]. For supershift experiments nuclear extracts were pre-incubated with 2 μg of C/EBPα antibody (N19x and 14AAx, Santa Cruz) or the respective isotype control on ice for 20 min before adding labelled oligonucleotides. As a control, the C/EBP binding site derived from the IL-6 promoter (5’-GGACGTCACATTGCACAATCTTAATAA-3’) [45] was used. Binding sites are underlined, mutated nucleotides are indicated in bold.

### Chromatin immunoprecipitation (ChIP) assay

HPV 8E6 expressing and pLXSN control HaCaT cells were seeded at 3 x 10^6^ cells per 10-cm dish and used for ChIP assay according to the protocol of the respective kit from Abcam. Briefly, cells were treated with formaldehyde at a final concentration of 0.75% and sonicated 4 times for 7 min each using a Diagenode Bioruptor (Diagenode). The cleared supernatant was incubated for 1 h at 4˚C with 3 μg of p300 monoclonal RW128 mouse antibody (Millipore) or mouse IgG1 (New England BioLabs). The immune complexes were precipitated with 60 μl protein A/G Sepharose (Santa Cruz Biotechnology) in the presence of salmon sperm DNA overnight at 4˚C. After elution of the protein-DNA complexes and reversing the cross-links, DNA was isolated and the C/EBPα regulatory region was detected and quantified by real-time PCR using the 5’-TAAGGCCACTGTCGGTGAAG-3’ and 5`-GAGCCCTCAAGTGTCTCCTG-3`primers and PowerUp SYBR Green (ThermoFisher Scientific). Results were normalized to isotype control.

### Statistical analysis

To evaluate statistical differences between two groups, a two-sided, unpaired *t* test was applied using GraphPad Prism 6. ****p<0.0001, ***p<0.001, **p<0.01, *p<0.05, ns: not significant.

## Supporting information

S1 Fig HPV8 oncogene expression in retrovirally infected NHK. NHK stably expressing HPV8 E6, E7, E6/E7 or the corresponding pLXSN control cells were analyzed for **(A)** HPV8 E6 mRNA or **(B)** E7 mRNA expression by qRT-PCR in relation to RPL13A.
(TIF)

S2 Fig HPV8 oncogene expression in retrovirally infected HaCaT and quantification of Western and Northern blots. HaCaT cells stably expressing HPV8 E6 or the corresponding pLXSN control cells were analyzed for **(A)** HPV8 E6 mRNA expression by qRT-PCR in relation to RPL13A and **(B)** p63 protein expression by Western blot in relation to actin expression. Three independent experiments were summarized. **(C)** Quantification of miR-203 expression determined by three independent Northern blot experiments in relation to 28SRNA. Cells were stimulated with 1.2 mM calcium for 72 h. (*p<0.05)
(TIF)

S3 Fig Quantification of Western blots and IHC staining. 10 nM p63-specific siRNA (or si-control) **(A, D)** or 20 nM hsa-miR-203 (or control-mimic) **(B, E)** were transfected in HPV8 E6 expressing and pLXSN control HaCaT cells. 48 h or 72 h post transfection, p63 **(A, B)** or involucrin **(D, E)** protein expression levels were determined by Western blot and quantified in relation to actin expression. Three independent experiments were summarized. **(C)** Involucrin IHC staining from three independent organotypic cultures generated from HPV8 E6 expressing or the corresponding pLXSN control cells were quantified for p63-positive nuclei. (ns: not significant, *p<0.05, **p<0.01, ***p<0.001, ****p<0.0001)
(TIF)

S4 Fig C/EBPα and p300 protein knock-down. **(A)** Organotypic cultures generated from HaCaT cells transfected with 10 nM single siRNAs directed against C/EBPα (or si-control) were stained for C/EBPα expression (red). IRS score and knock-down efficiencies are indicated. **(B)** NHK cells were transfected with 10 nM single siRNAs or a siRNA pool directed against p300 (or si-control) and harvested after 48 h for protein extracts. p300 expression was investigated by Western blot with p300-specific antibody (RW128). Actin served as loading control.
(TIF)

S5 Fig C/EBPα and C/EBPβ mRNA expression in retrovirally infected NHK. NHK stably expressing HPV8 E6 or the corresponding pLXSN control cells were stimulated with **(A)** 50 ng/ml PMA for 24 h or **(B)** 1.2 mM Ca^2+^ for 72 h. C/EBPα mRNA expression was analyzed by qRT-PCR in relation to RPL13A. Data from pLXSN cells are the same as presented in Fig 5D and 5E. **(C)** C/EBPβ mRNA expression was analyzed by qRT-PCR in NHK expressing HPV8 E6 and/or E7. The mean values ± SD from *n* ≥ 3 experiments performed in duplicates are shown. (ns: not significant, *p<0.05, **p<0.01, ***p<0.001, ****p<0.0001)
(TIF)

S6 Fig p63 expression in productive EV-lesion. Sections of HPV8-positive lesional skin with cytopathic effects were stained using antibodies against p63 (red). Shown is the same patient as in Fig 7B and 7E.
(TIF)

S7 Fig JunB expression in NHK and effect of MAML1 on C/EBPα mRNA expression. **(A)** NHK stably expressing HPV8 E6, Δ8E6 (aa Δ132–136) or the corresponding pLXSN cells were analyzed in qRT-PCR for JunB expression as described. **(B)** NHK cells were transfected with 10 nM single siRNAs or a siRNA pool directed against p300 (or si-control) and harvested after 48 h. mRNA expression of JunB was determined by qRT-PCR in relation to RPL13A. **(C)** NHK were transfected with 10 nM MAML1-specific siRNA (or si-control), harvested 48 h later and mRNA expression of C/EBPα and MAML1 were determined by qRT-PCR in relation to RPL13A. The mean values ± SD from *n* ≥ 3 experiments performed in duplicates are shown. (ns: not significant, *p<0.05, **p<0.01, ****p<0.0001).
(TIF)

S1 Table siRNA and hsa-miR-203 mimic sequences. (PDF)

S2 Table qRT-PCR oligonucleotide sequences and corresponding UPL hydrolysis probes from Roche Diagnostics. (PDF)

S3 Table Knock-down efficiencies of siRNA experiments. Quantification by qRT-PCR (% reduction of mRNA expression in comparison to si-control).
(PDF)
