# Supplementary figures and images for: Identification of C/EBPα as a novel target of the HPV8 E6 protein regulating miR-203 in human keratinocytes
Source: PLoS Pathog. 2017 Jun 22;13(6):e1006406. doi: 10.1371/journal.ppat.1006406 (PMC5481020; doi:10.1371/journal.ppat.1006406)

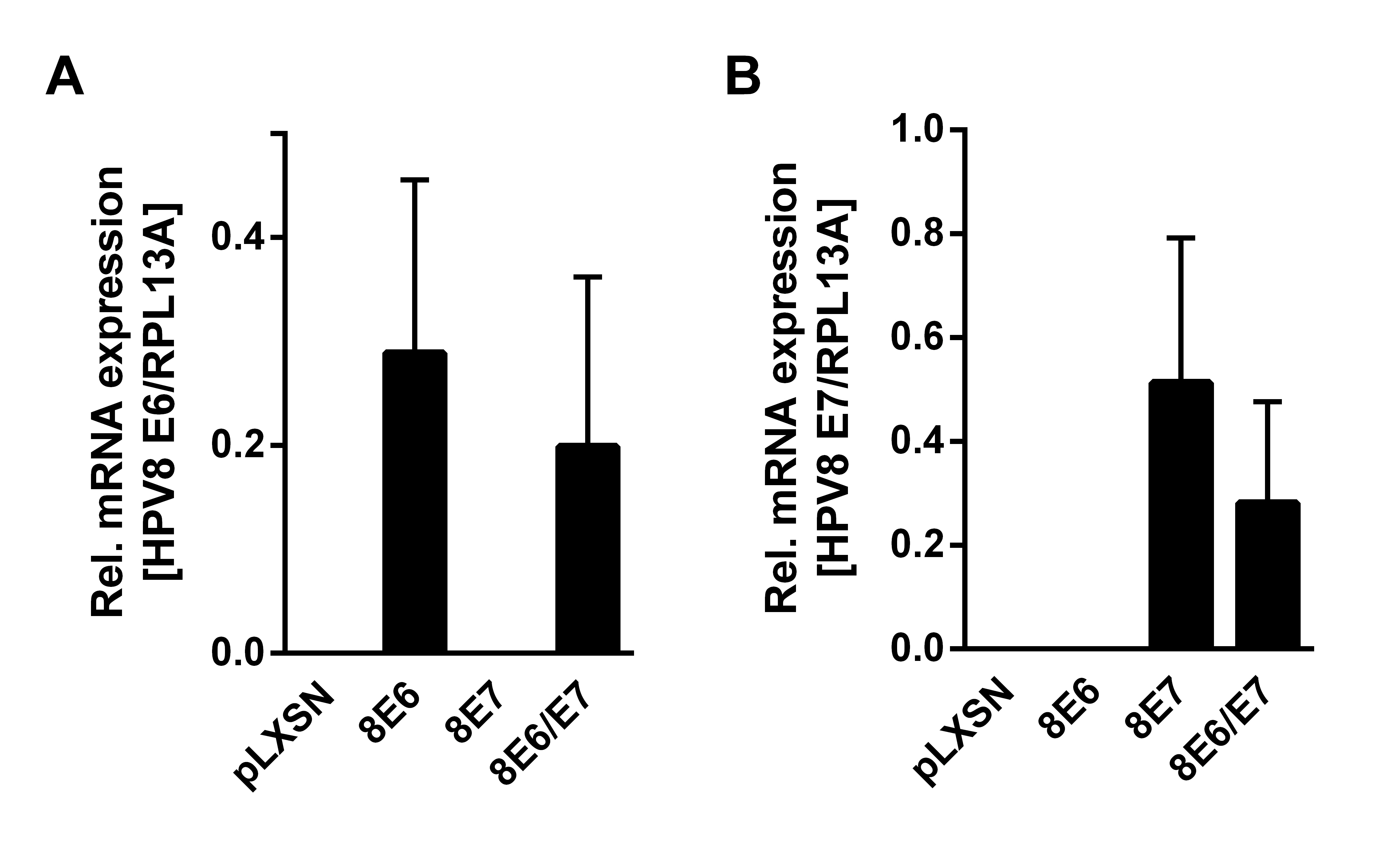

Supplement: S1 Fig — NHK stably expressing HPV8 E6, E7, E6/E7 or the corresponding pLXSN control cells were analyzed for (A) HPV8 E6 mRNA or (B) E7 mRNA expression by qRT-PCR in relation to RPL13A. (TIF) [file ppat.1006406.s001.tif]

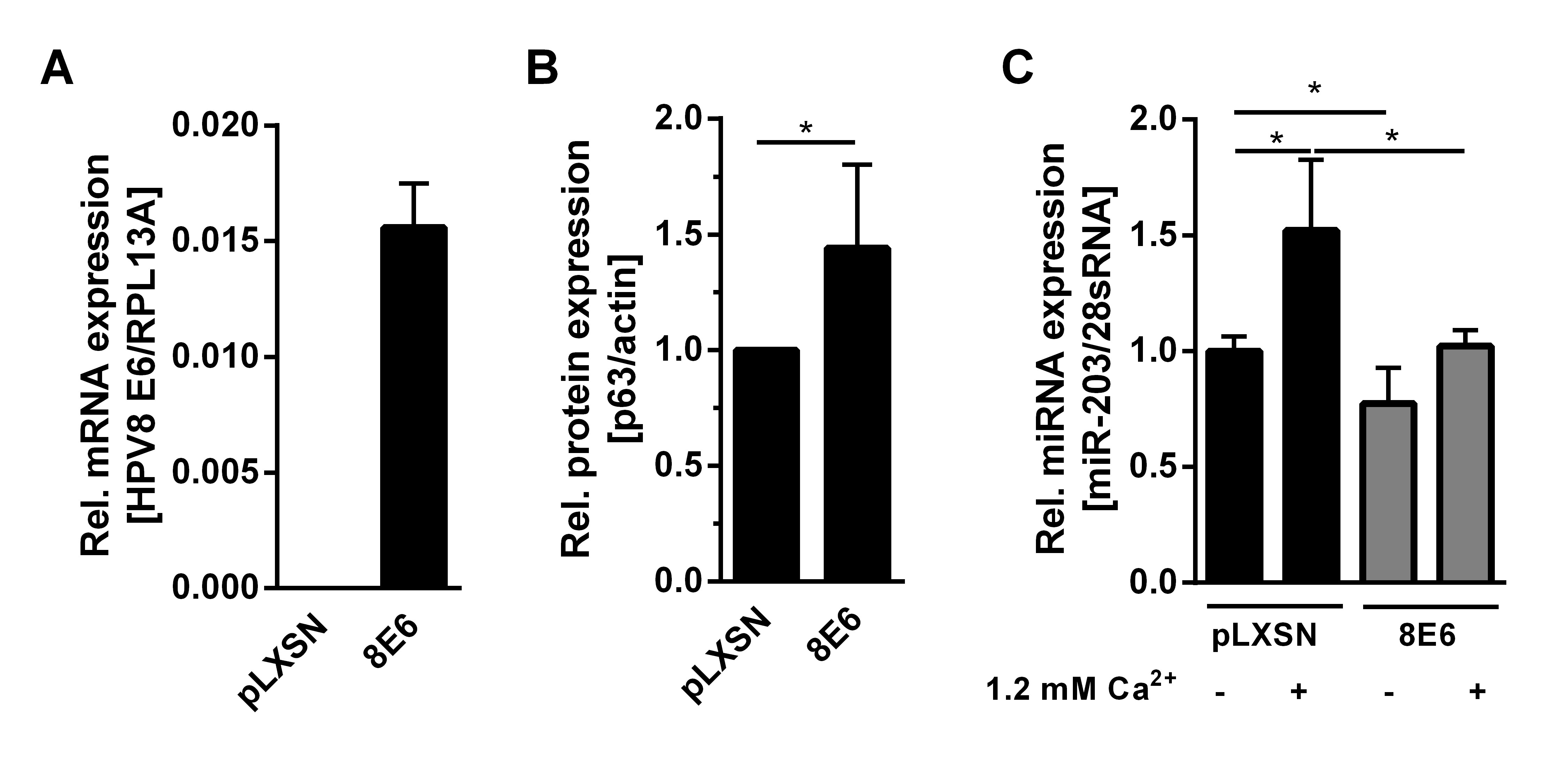

Supplement: S2 Fig — HaCaT cells stably expressing HPV8 E6 or the corresponding pLXSN control cells were analyzed for (A) HPV8 E6 mRNA expression by qRT-PCR in relation to RPL13A and (B) p63 protein expression by Western blot in relation to actin expression. Three independent experiments were summarized. (C) Quantification of miR-203 expression determined by three independent Northern blot experiments in relation to 28SRNA. Cells were stimulated with 1.2 mM calcium for 72 h. (*p<0.05) (TIF) [file ppat.1006406.s002.tif]

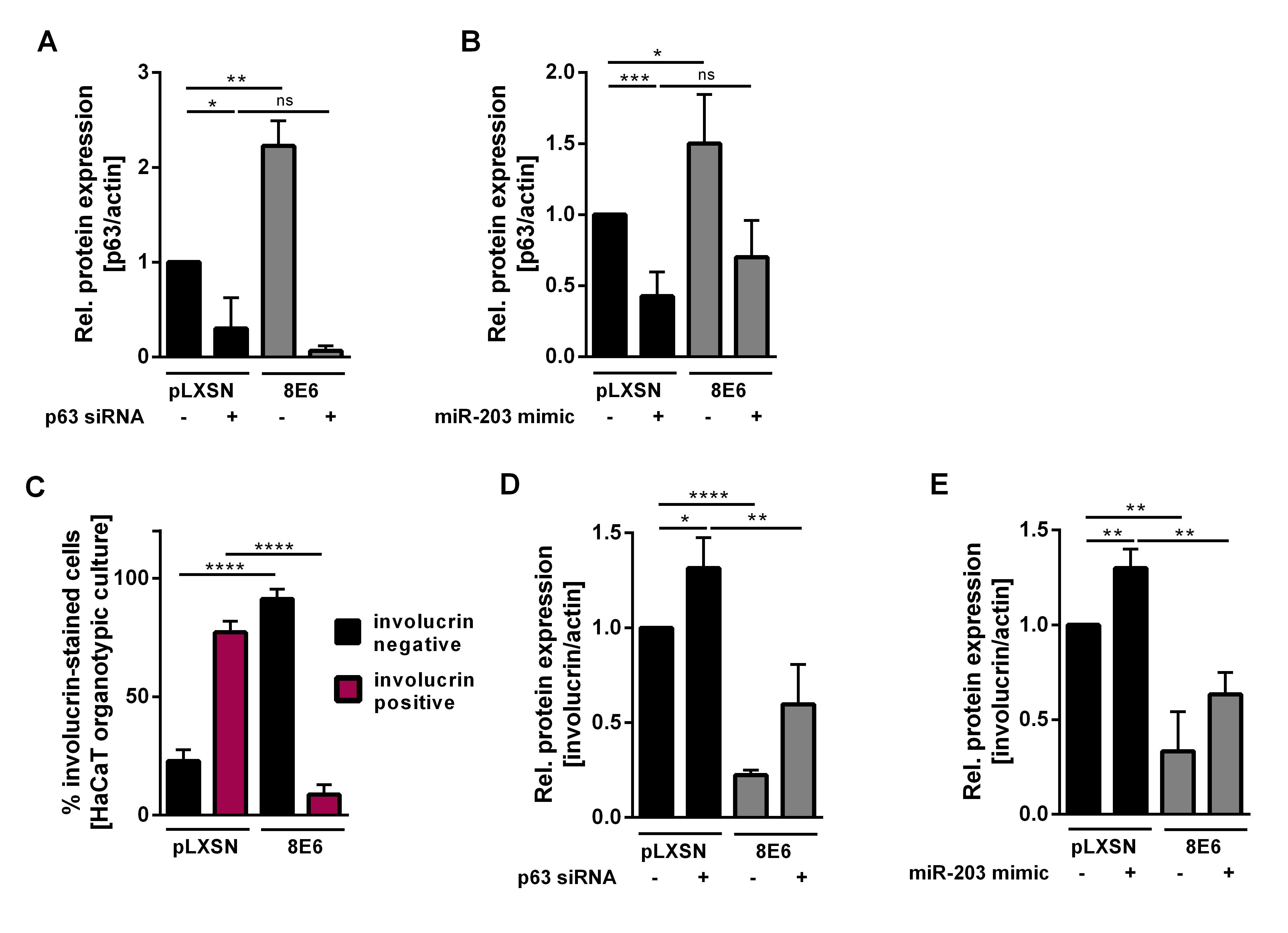

Supplement: S3 Fig — 10 nM p63-specific siRNA (or si-control) (A, D) or 20 nM hsa-miR-203 (or control-mimic) (B, E) were transfected in HPV8 E6 expressing and pLXSN control HaCaT cells. 48 h or 72 h post transfection, p63 (A, B) or involucrin (D, E) protein expression levels were determined by Western blot and quantified in relation to actin expression. Three independent experiments were summarized. (C) Involucrin IHC staining from three independent organotypic cultures generated from HPV8 E6 expressing or the corresponding pLXSN control cells were quantified for p63-positive nuclei. (ns: not significant, *p<0.05, **p<0.01, ***p<0.001, ****p<0.0001) (TIF) [file ppat.1006406.s003.tif]

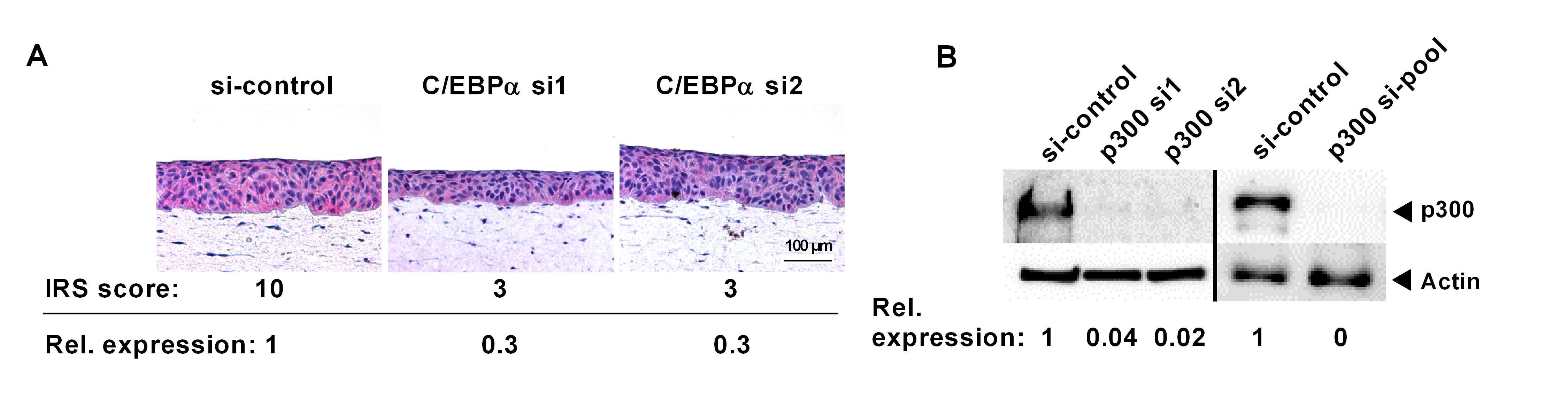

Supplement: S4 Fig — (A) Organotypic cultures generated from HaCaT cells transfected with 10 nM single siRNAs directed against C/EBPα (or si-control) were stained for C/EBPα expression (red). IRS score and knock-down efficiencies are indicated. (B) NHK cells were transfected with 10 nM single siRNAs or a siRNA pool directed against p300 (or si-control) and harvested after 48 h for protein extracts. p300 expression was investigated by Western blot with p300-specific antibody (RW128). Actin served as loading control. (TIF) [file ppat.1006406.s004.tif]

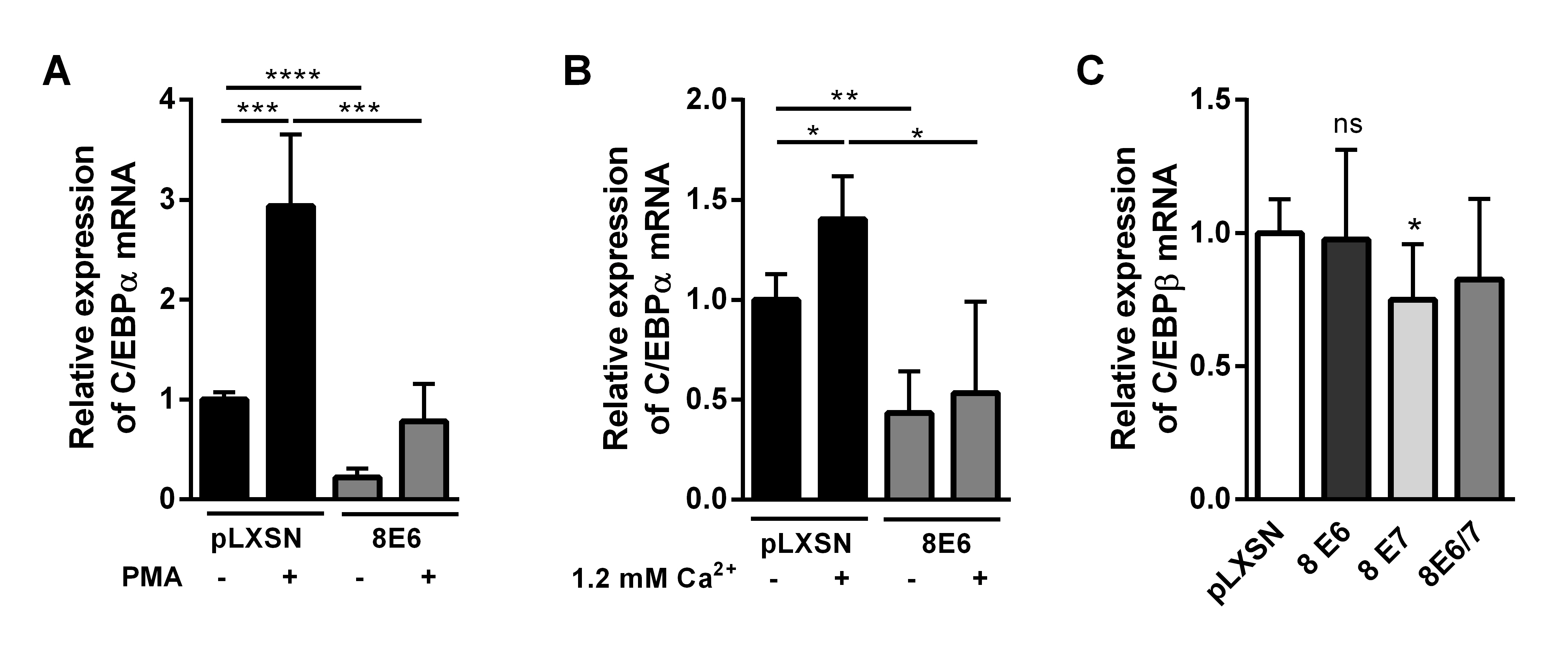

Supplement: S5 Fig — NHK stably expressing HPV8 E6 or the corresponding pLXSN control cells were stimulated with (A) 50 ng/ml PMA for 24 h or (B) 1.2 mM Ca2+ for 72 h. C/EBPα mRNA expression was analyzed by qRT-PCR in relation to RPL13A. Data from pLXSN cells are the same as presented in Fig 5D and 5E. (C) C/EBPβ mRNA expression was analyzed by qRT-PCR in NHK expressing HPV8 E6 and/or E7. The mean values ± SD from n ≥ 3 experiments performed in duplicates are shown. (ns: not significant, *p<0.05, **p<0.01, ***p<0.001, ****p<0.0001) (TIF) [file ppat.1006406.s005.tif]

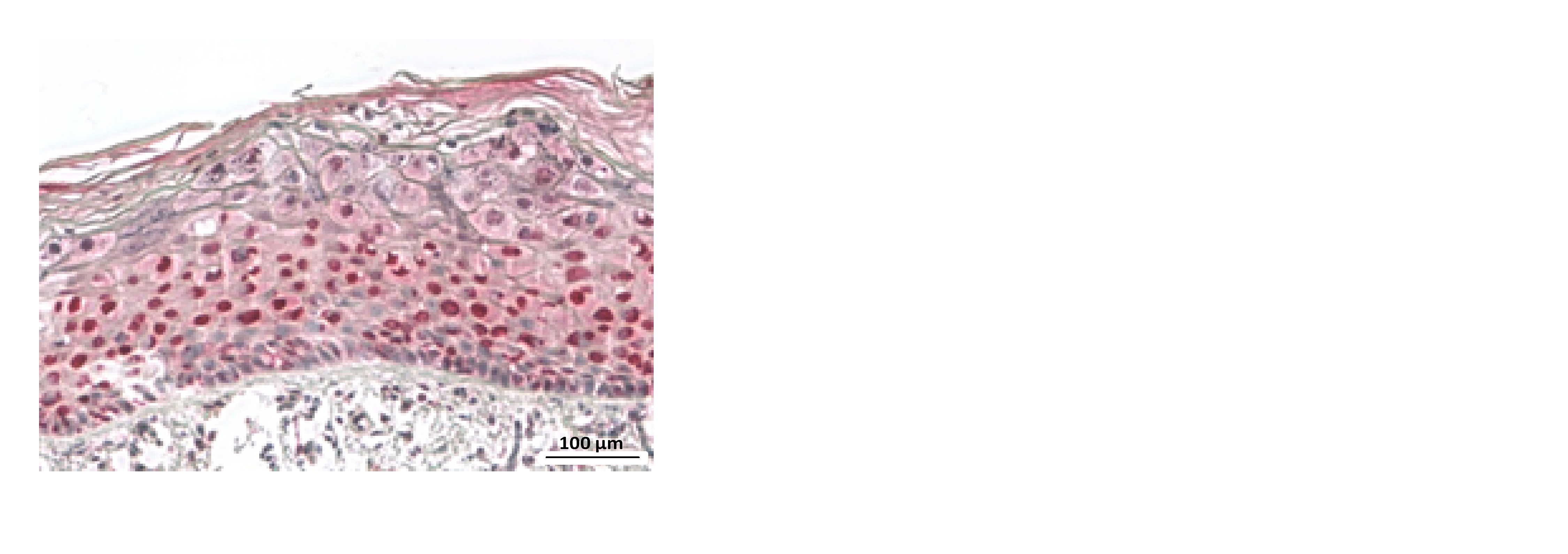

Supplement: S6 Fig — Sections of HPV8-positive lesional skin with cytopathic effects were stained using antibodies against p63 (red). Shown is the same patient as in Fig 7B and 7E. (TIF) [file ppat.1006406.s006.tif]

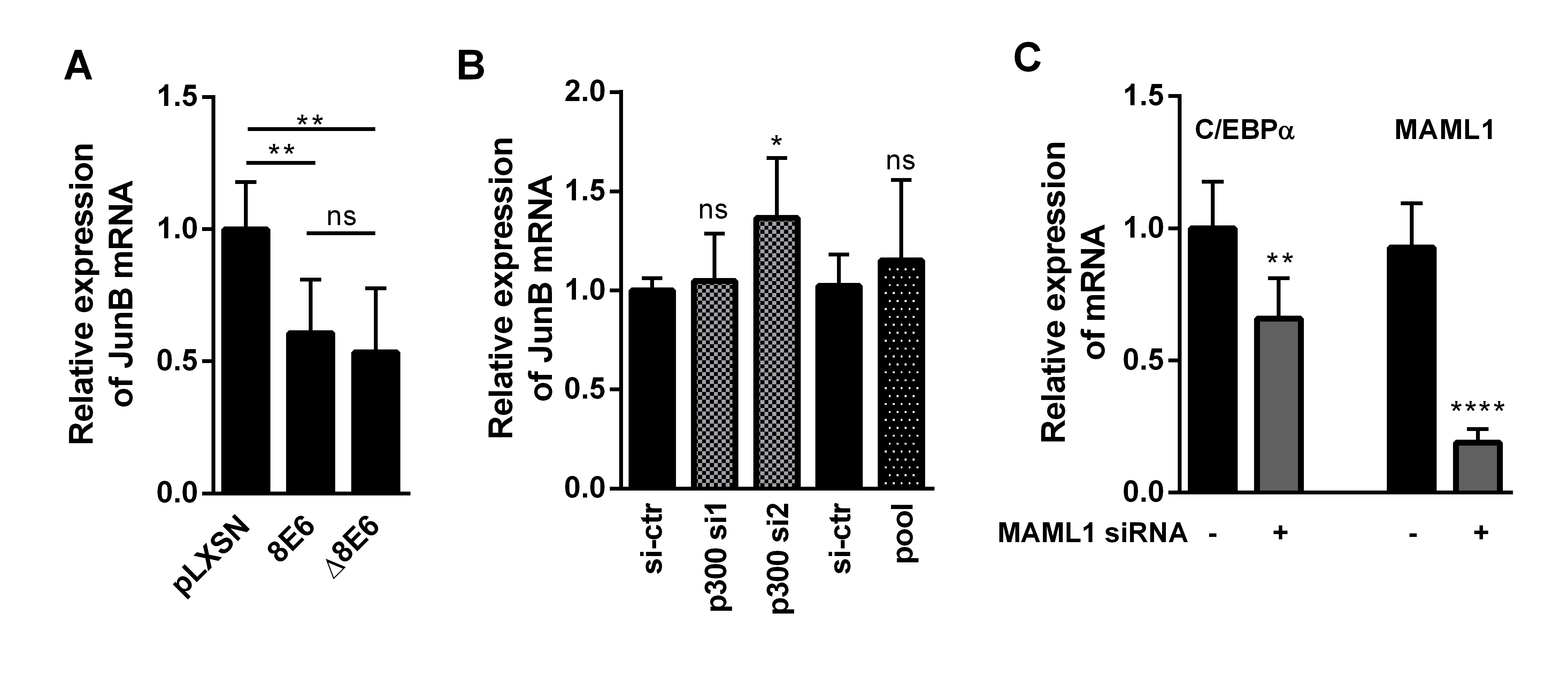

Supplement: S7 Fig — (A) NHK stably expressing HPV8 E6, Δ8E6 (aa Δ132–136) or the corresponding pLXSN cells were analyzed in qRT-PCR for JunB expression as described. (B) NHK cells were transfected with 10 nM single siRNAs or a siRNA pool directed against p300 (or si-control) and harvested after 48 h. mRNA expression of JunB was determined by qRT-PCR in relation to RPL13A. (C) NHK were transfected with 10 nM MAML1-specific siRNA (or si-control), harvested 48 h later and mRNA expression of C/EBPα and MAML1 were determined by qRT-PCR in relation to RPL13A. The mean values ± SD from n ≥ 3 experiments performed in duplicates are shown. (ns: not significant, *p<0.05, **p<0.01, ****p<0.0001). (TIF) [file ppat.1006406.s007.tif]
