# Supplementary material for: Identification of C/EBPα as a novel target of the HPV8 E6 protein regulating miR-203 in human keratinocytes
Source: PLoS Pathog. 2017 Jun 22;13(6):e1006406. doi: 10.1371/journal.ppat.1006406 (PMC5481020; doi:10.1371/journal.ppat.1006406)
Supplement: S1 Table — (PDF) [file ppat.1006406.s008.pdf]

S1 Table. siRNA and hsa-miR-203 mimic sequences. (Dharmacon, Freiburg, Germany)

siRNA as indicated in figure	siRNA	Target sequence
si-control	ON-TARGETplus Control Non-targeting siRNA#2	5'-ugguuuacauguuguguga-3'
p300 si pool	p300 ON-TARGETplus SMARTpool	5'-ggacuacccuaucaaguaa-3'<br>5'-gacaagggauaaugccuaa-3'<br>5'-guucaauaaugccugguua-3'<br>5'-cgacagggaugcagcaaca-3'
p300 si1	p300 ON-TARGETplus 1	5'-guucaauaaugccugguua-3'
p300 si2	p300 ON-TARGETplus 2	5'-cgacagggaugcagcaaca-3'
C/EBPα si pool	C/EBPα ON-TARGETplus SMARTpool	5'-acaaugaccgccugcgcaa-3'<br>5'-cacgagacguccaucgaca-3'<br>5'-gaacagcugagccgcgaac-3'<br>5'-gaacagcaacgaguaccgg-3'
C/EBPα si1	C/EBPα ON-TARGETplus 1	5'-cacgagacguccaucgaca-3'
C/EBPα si2	C/EBPα ON-TARGETplus 2	5'-gaacagcugagccgcgaac-3'
p63 si	p63 ON-TARGETplus SMARTpool	5'-gaugaacuguuauacuuac-3'<br>5'-cgacagucuuguacaauuu-3'<br>5'-gcacacagacaaaugaauu-3'<br>5'-ucuaucagauugagcauua-3'
miR-203 mimic	miRDIAN hsa-miR-203 mimic	5'-gugaaauguuuaggaccacuag-3'
control-mimic	miRDIAN microRNA Mimic Negative control #1	unknown
