# Supplementary material for: Identification of C/EBPα as a novel target of the HPV8 E6 protein regulating miR-203 in human keratinocytes
Source: PLoS Pathog. 2017 Jun 22;13(6):e1006406. doi: 10.1371/journal.ppat.1006406 (PMC5481020; doi:10.1371/journal.ppat.1006406)
Supplement: S2 Table — (PDF) [file ppat.1006406.s009.pdf]

S2 Table. qRT-PCR oligonucleotide sequences and corresponding UPL hydrolysis probes from Roche Diagnostics.

mRNA	Sequence	UPL-Probe
C/EBPα	5'- GTGGACAAGAACAGCAACGA-3'<br>5'- CACTGGTCAGCTCCAGCAC-3'	#84
C/EBPβ	5'- CTGGAGACGCAGCACAAG-3'<br>5'- ACAGCTGCTCCACCTTCTTC-3'	#1
ΔNp63α	5'- GGAAAACAATGCCCAGACTC-3'<br>5'- CTGCTGGTCCATGCTGTTC-3'	#45
Involucrin	5'- AAGGGATCAGCAGCTAAACAAA-3'<br>5'- TTGATCCAGTTGCTGGTCTAAG-3'	#11
MAML	5'- TATGCCTTTCCGATCACTGG-3'<br>5'- CTGGGTCCCAACACTGGTAG-3'	#68
p300	5'- CAAGCAAAGAACCAGCAGAA-3'<br>5'- CATTTACTCCCATAGGACTAGCACTC-3'	#64
RPL13A	5'- AGCGGATGAACACCAACC-3'<br>5'- TTTGTGGGGCAGCATACTC-3'	#28
