# Supplementary material for: Identification of C/EBPα as a novel target of the HPV8 E6 protein regulating miR-203 in human keratinocytes
Source: PLoS Pathog. 2017 Jun 22;13(6):e1006406. doi: 10.1371/journal.ppat.1006406 (PMC5481020; doi:10.1371/journal.ppat.1006406)
Supplement: S3 Table — Quantification by qRT-PCR (% reduction of mRNA expression in comparison to si-control). (PDF) [file ppat.1006406.s010.pdf]

S3 Table. Knock-down efficiencies of siRNAs quantified by qRT-PCR (% reduction of mRNA expression in comparison to si-control).

	**siRNA 1**	**siRNA 2**	**pool**
p63	-	-	70%
p300	81%	65%	60%
C/EBPα	72%	80%	52%
